# Simultaneous optimization of lignocellulosic sugar catabolism via systematic laboratory evolution under complex selection pressure

**DOI:** 10.1038/s41467-026-75974-x

**Published:** 2026-07-29

**Authors:** Sunghwa Woo, Hyun Gyu Lim, Brenna Norton-Baker, Torrey M. Lind, Nathan E. Gladden, Yan Chen, Ciara de Venecia, Thomas Eng, Christopher W. Johnson, Aindrila Mukhopadhyay, Christopher J. Petzold, Adam M. Guss, Gregg T. Beckham, Adam M. Feist

**Affiliations:** 1 Department of Bioengineering, University of California San Diego, La Jolla, CA USA; 2 Joint BioEnergy Institute, Emeryville, CA USA; 3 Department of Biological Sciences and Bioengineering, Inha University, Incheon, South Korea; 4 Institute of AI-driven Industrial Biotechnology, Inha University, Incheon, South Korea; 5 Renewable Resources and Enabling Sciences Center, National Laboratory of the Rockies, Golden, CO USA; 6 Agile BioFoundry, Emeryville, CA USA; 7 Biosciences Division, Oak Ridge National Laboratory, Oak Ridge, TN USA; 8 Biological Systems and Engineering Division, Lawrence Berkeley National Laboratory, Berkeley, CA USA; 9 BRIGHT, Technical University of Denmark, Lyngby, Denmark

**Keywords:** Experimental evolution, Metabolic engineering, Applied microbiology

## Abstract

Efficient co-utilization of hexose and pentose sugars from lignocellulose is essential for microbial bioconversion, yet engineered catabolic pathways can be unstable or suboptimal in complex resource environments. Here, we use a *Pseudomonas putida* strain engineered to catabolize xylose and arabinose to examine how resource abundance, temporal availability, and subculturing shape evolutionary outcomes. Using an automated adaptive laboratory evolution (ALE) platform, we evolve the strain under simple single-substrate and complex multi-substrate selection pressures. These environments drive divergence between catabolic specialists and generalists. Weak or absent selection for xylose frequently leads to loss of xylose catabolism, whereas carbon-limited mixed-sugar environments promote stable retention and coordinated optimization of multiple catabolic pathways, enhancing growth and substrate utilization. Genomic, proteomic, and biochemical analyses show that pathway-specific fitness costs determine evolutionary stability. A generalist clone also shows improved indigoidine production from mixed sugars relative to the parental strain. Together, these findings show how resource dynamics shape fitness landscapes that govern catabolic specialization, generalization, evolutionary trade-offs, and engineering of bioconversion.

## Abstract (web summary)

Efficient co-utilization of sugars from lignocellulose is essential for microbial bioconversion. Here the authors perform laboratory evolution of *P. putida* to reveal how selection shapes retention or loss of catabolic pathways, offering design rules for biomanufacturing phenotypes.

## Introduction

A major challenge in lignocellulosic bioconversion is enabling microbes to efficiently assimilate mixed sugars under industrially relevant conditions. Lignocellulosic hydrolysates contain both hexoses and pentoses, and biocatalysts must co-utilize these substrates to maximize production yields and rates^1–4^. Recent advances in industrial biotechnology have enhanced the efficiency of microbial biocatalysts for converting biomass feedstock into value-added chemical products^5,6^. Nevertheless, the engineering of such microbes is complex, given the regulatory and metabolic strategies that microbes have evolved to handle fluctuating substrate availability and ecological competition in natural environments^7–9^. As a result, microbial species often exhibit distinct substrate preferences, and hierarchical substrate utilization is governed by mechanisms such as carbon catabolite repression, which promotes rapid growth and competitive dominance by prioritizing preferred substrates^10–12^. While advantageous in natural ecosystems, such regulatory mechanisms hinder the simultaneous assimilation of mixed substrates, which is a critical limitation for microbial biocatalysts designed to process biomass-derived feedstocks^13–15^.

*Pseudomonas putida*, particularly the KT2440 strain, has emerged as an attractive and well-established microbial host for bioproduction, owing to its stress tolerance and ability to catabolize aromatic compounds^16–18^. Engineering efforts have expanded its substrate range through the introduction of multiple variants of enabling catabolic pathways^19–26^. Building on these advances, the JE3692 strain, a derivative of *P. putida* KT2440, enabled simultaneous utilization of multiple substrates, including glucose, xylose, arabinose, acetate, and *p*-coumarate, via chromosomally integrated pentose catabolic pathways, adaptive laboratory evolution (ALE), and alleviation of carbon catabolite repression^23^. Despite this progress, fully optimizing the coordinated utilization of multiple sugars, especially non-native substrates, remains difficult due to complex regulatory and metabolic interplay.

ALE is a powerful approach to strain optimization that can help overcome strain inefficiencies via diversity generation and selection^27^. Most ALE studies to date have been conducted under simple selection pressure with a single substrate^28–33^. Such regimes may not impose sufficient or appropriate selection pressure for performance in mixed-sugar contexts, as catabolic pathways not under direct selection often remain unoptimized. Moreover, heterologous or engineered pathways that reduce fitness can be lost, unless their expression is directly coupled to survival. While some studies have attempted co-optimization under multiple selection pressures within a single ALE campaign, outcomes have typically included either limited fitness improvements compared to single-substrate ALE or the loss of substrate-specific beneficial mutations^34,35^. This underscores the need for alternative ALE strategies that incorporate environmental complexity and dynamics to drive balanced optimization of multiple substrates.

Here, we use an automated ALE platform to evolve the previously engineered *P. putida* strain, JE3692, carrying heterologous xylose and arabinose catabolic pathways^23^ under both single-sugar and multiple-sugar regimes. This comparative approach allows us to probe how environmental composition and temporal dynamics shape evolutionary trajectories in an application-driven context. Multi-omics analyses reveal distinct adaptive strategies, including regulatory rewiring and selective pathway retention. Furthermore, we show that a strain with improved mixed-sugar utilization exhibits enhanced production of the non-ribosomal peptide indigoidine, underscoring the biotechnological potential of evolution-guided strategies. Our findings demonstrate that multi-substrate ALE regimes dissect carbon catabolism by revealing how differential substrate utilization rates, pathway-specific fitness costs, and time-varying selection pressures shape evolutionary outcomes, providing design guidelines for engineering microbial platforms for mixed and heterogeneous feedstocks as well as similar complex evolutionary engineering goals.

## Results

### Systematic evolution under complex selection pressure for optimized utilization of major lignocellulosic sugars

**Fig. 1:**
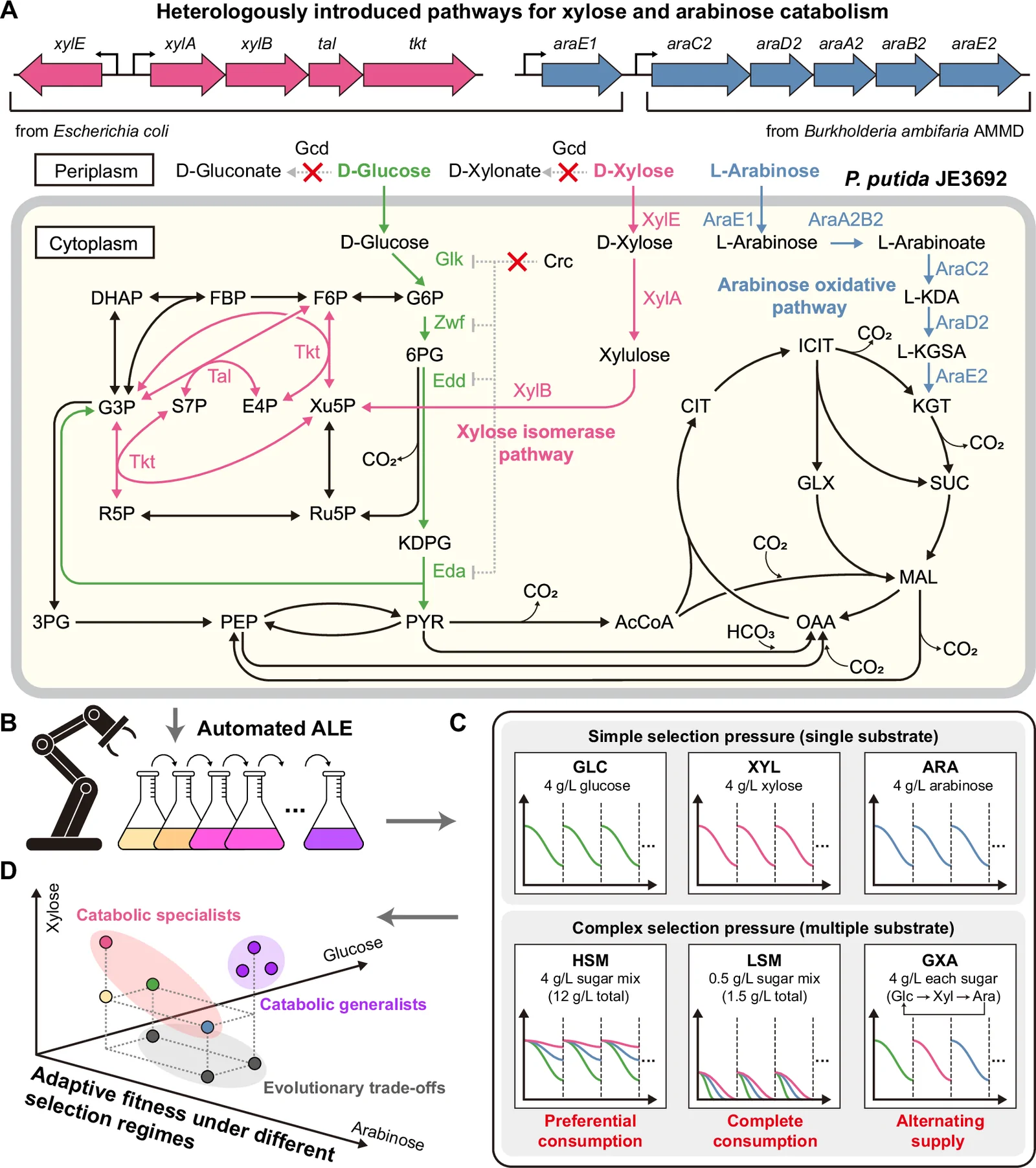
Systematic evolution under complex selection pressure for optimized utilization of major lignocellulosic sugars. **A** Catabolic pathways for glucose, xylose, and arabinose in JE3692. Genes for each pathway are highlighted with a different color: green, endogenous Entner-Doudoroff (ED) pathway; pink, heterologous xylose isomerase pathway; blue, heterologous arabinose oxidative pathway. Gray dotted lines and red crosses indicate the deleted genes (*gcd* and *crc*). G6P D-glucose 6-phosphate, 6PG D-gluconate 6-phosphate, KDPG 2-dehydro-3-deoxy-D-gluconate 6-phosphate, F6P D-fructose 6-phosphate, FBP D-fructose 1,6-bisphosphate, DHAP dihydroxyacetone phosphate, G3P D-glyceraldehyde 3-phosphate, 3PG 3-phospho-D-glycerate, PEP phosphoenolpyruvate, PYR pyruvate, AcCoA acetyl-CoA, CIT citrate, ICIT isocitrate, KGT 2-ketoglutarate, SUC succinate, MAL malate, OAA oxaloacetate, GLX glyoxylate, Xu5P xylulose 5-phosphate, Ru5P ribulose 5-phosphate, R5P ribose 5-phosphate, E4P D-erythrose 4-phosphate, S7P D-sedoheptulose 7-phosphate, L-KDA 2-keto-3-deoxy-L-arabinonate, L-KGSA 2-ketoglutarate semialdehyde. **B** Automated ALEbot robotic platform, which enabled consistent, systematic, and parallel execution of multiple independently controlled evolution lineages, thereby maximizing consistency. **C** ALE strategies used in this study. Simple selection pressures (single substrate): GLC (glucose 4 g/L), XYL (xylose 4 g/L), ARA (arabinose 4 g/L). Complex selection pressures (multiple substrates): mixed-substrate regimes including HSM (glucose 4 g/L, xylose 4 g/L, and arabinose 4 g/L; selective sugar consumption allowed) and LSM (glucose 0.5 g/L, xylose 0.5 g/L, and arabinose 0.5 g/L; complete sugar consumption mandated), and an alternating-substrate regime, GXA (glucose 4 g/L, xylose 4 g/L, or arabinose 4 g/L; each sugar supplied sequentially). Each ALE was performed with four replicates (*n* = 4). The differently colored curves in the simplified graph represent the expected sugar consumption profiles for each passage, separated by dotted lines: green for glucose, pink for xylose, and blue for arabinose. **D** A conceptual diagram of the fitness landscape highlighting potential evolutionary outcomes. Evolutionary outcomes may range from catabolic specialists, optimized for catabolism of a specific substrate but often incurring trade-offs on others, to catabolic generalists, which utilize a range of targeted metabolites without significant trade-offs on any one substrate.

The engineered *Pseudomonas putida* strain JE3692 was used as the starting strain for all ALE campaigns^23^. This strain natively utilizes glucose and was previously modified to catabolize xylose and arabinose, two additional major sugars commonly derived from hemicellulose (Fig. 1A). This strain harbors two heterologous catabolic pathways: for xylose utilization, the xylose isomerase pathway from *Escherichia coli* includes *xylE*, *xylA*, *xylB*, *tkt*, and *tal*. For arabinose metabolism, the oxidative arabinose pathway includes *araE1* from *E. coli* and *araA2*, *araB2*, *araC2*, *araD2*, and *araE2* from *Burkholderia ambifaria* AMMD^36^. In the JE3692 strain, two deletions were introduced: *gcd* (glucose dehydrogenase) was deleted to prevent the formation of gluconate and xylonate^21,37^, and *crc* (catabolite repression control protein) was deleted to avoid the native regulation for sugar catabolism^14,25,38^. This strain represented both a promising candidate to build upon for industrial bioprocessing and a relevant test case for exploring ALE-driven optimization under complex selection pressure, given its combination of native and engineered metabolic capabilities.

The baseline fitness of JE3692 was evaluated in single-sugar and mixed-sugar conditions. The strain exhibited a specific growth rate of 0.42 ± 0.04 h^−1^ in glucose 4 g/L minimal medium, while growth rates in xylose 4 g/L and arabinose 4 g/L minimal media were lower, at 0.20 ± 0.01 and 0.14 ± 0.02 h^−1^, respectively. In a mixed-sugar minimal medium (4 g/L each), the strain grew at 0.46 ± 0.04 h^−1^, a modestly lower rate than previously reported (0.60 h^−1^), likely due to differences in cultivation conditions, such as sugar composition, aeration, and vessel type^23^. Although JE3692 was capable of simultaneously utilizing glucose, xylose, and arabinose in mixed-sugar media, the lower growth rates on xylose and arabinose relative to glucose highlight differences in catabolic efficiency among the pathways and motivate further optimization of the heterologous sugar utilization systems^25^.

To investigate variation in evolutionary adaptation across different sugar conditions and to enable robust, multi-sugar conversion^34^, we performed multiple ALE experiments under distinct sugar concentrations and transfer criteria. All ALE experiments were conducted using an automated robotic ALEbot platform, which enabled consistent, systematic, and parallel execution of multiple, independently controlled evolution lines, thereby minimizing human error^39^ (Fig. 1B). The ALE strategies were categorized into simple and complex selection pressures (Fig. 1C and Supplementary Table 1). Under the simple selection pressures, modified M9 minimal medium was supplemented with 4 g/L of a single sugar, namely glucose (GLC), xylose (XYL), or arabinose (ARA). The complex selection pressures involved multiple substrates and consisted of three distinct regimes: HSM (High Sugar Mixture; 4 g/L each glucose, xylose, and arabinose) with OD-based transfer; LSM (Low Sugar Mixture; 0.5 g/L each) with sugar depletion-based transfer; and GXA, namely sequential exposure to glucose, xylose, and arabinose (4 g/L each). Within this category, GXA represented an alternating-substrate regime where substrate availability was intentionally modified between passages through automated process control. Conversely, HSM and LSM were categorized as mixed-substrate regimes where selection pressures underwent metabolism-driven temporal shifts within each individual passage, arising from the differential consumption rates of each sugar by the population. Each ALE condition was performed in quadruplicate, yielding 24 independent lineages.

ALE experiments were executed until growth improvements plateaued in at least three of the four replicates per condition. Across the ~3-month experiments, cultures underwent 63–109 passages, corresponding to 394–709 generations and 32.4–61.5 × 10^11^ cumulative cell divisions (CCDs) (Supplementary Table 2)^40^. Growth rate improvements were typically observed early and tended to stabilize after 34–55 passages (Supplementary Fig. 1). Clonal isolates were obtained from all end-point populations for phenotypic and genotypic characterization, as they were expected to exhibit distinct fitness landscapes depending on their respective selection regimes (Fig. 1D).

### Simple selection pressures drove substrate-specific catabolic adaptation and trade-offs in unselected functions

**Fig. 2:**
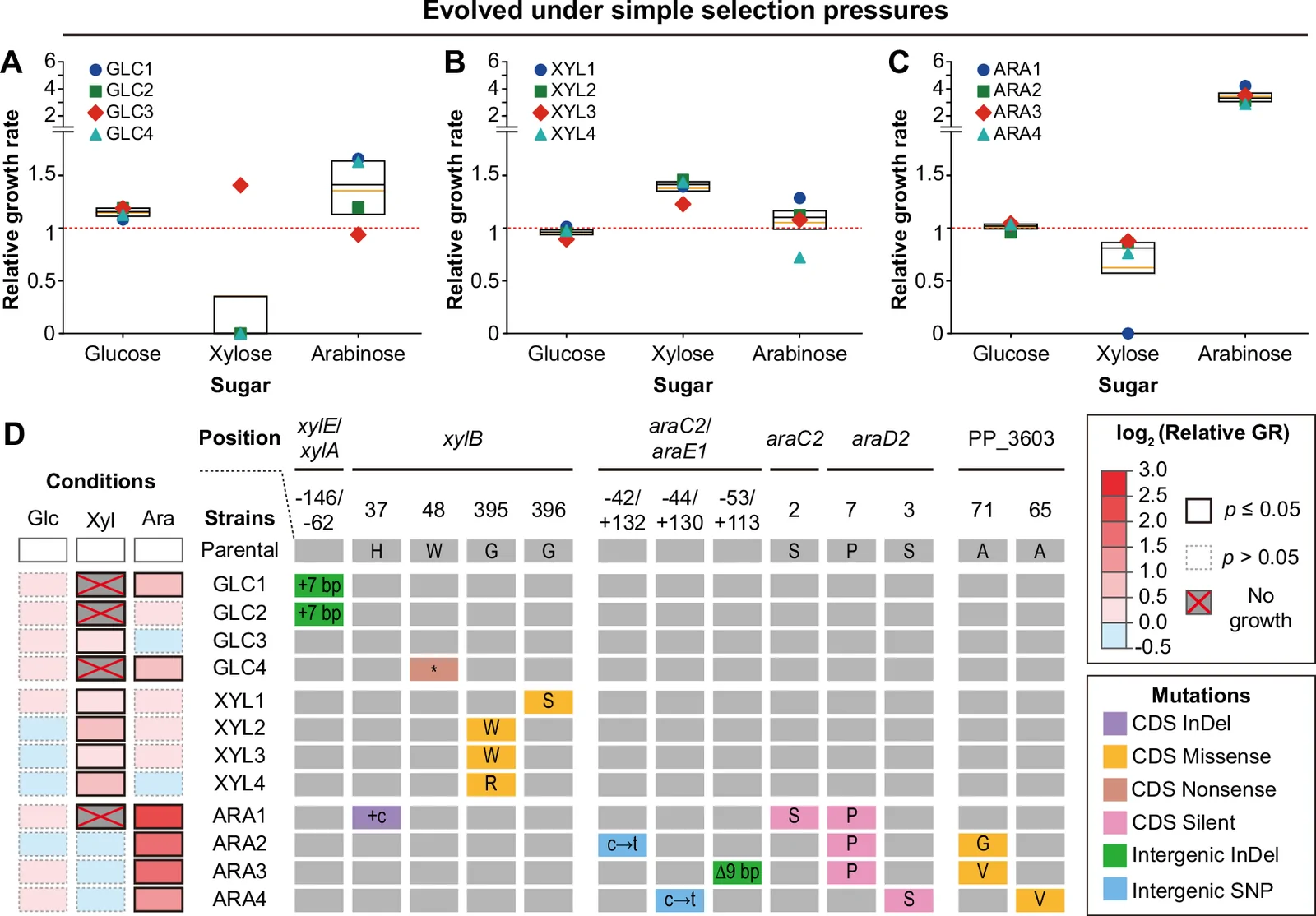
Sugar-specific adaptations and trade-offs in isolates evolved under simple selection pressures. Box plots show the relative growth rates of evolved strains isolated from **A** GLC, **B** XYL, and **C** ARA conditions under single-sugar conditions (glucose, xylose, and arabinose), compared to the starting JE3692 strain. Each dot represents the mean fold change in specific growth rate from duplicates (*n* = 2). Each box indicates the interquartile range, and each black and orange solid line in the box indicates the median and mean value, respectively. Red dotted lines indicate a fold change of 1. The *x*-axis represents sugar conditions under which the isolates were grown, and the *y*-axis represents the relative growth rate (fold change). **D** Detailed mutational analysis related to xylose and arabinose catabolism in isolates evolved under simple selection pressures. The right side shows mutations in the heterologous xylose and arabinose catabolic pathways. Rows and columns represent isolates and mutational positions, respectively. Uppercase and lowercase letters in boxes indicate amino acids and nucleobases, respectively. Different colors represent different mutation types detailed in the figure key. The heatmap on the left shows the log fold change in the specific growth rate of each evolved isolate compared to the parental strain under various sugar conditions, generated from the same dataset as (**A**–**C**). The rows and columns represent isolates and sugar conditions under which the isolates were grown, respectively. Boxes with the color scale represent a gradient according to the log fold change in the specific growth rate (relative GR), and gray boxes marked with cross indicate no growth. Source data are provided as a Source Data file.

The specific growth rates of isolates evolved under simple selection pressures were compared to assess their fitness improvements on each sugar. The GLC isolates showed an insignificant increase in growth rate in glucose minimal medium (*p* > 0.05) (Fig. 2A). The highest growth rate was 0.56 h^−1^, about 66% of that reported for evolved wild-type *P. putida* populations (0.85 h^−1^)^41^, likely reflecting disruption of the gluconate branch of glucose catabolism (*gcd* gene deletion). The XYL and ARA isolates exhibited improved growth when cultured in their respective selection conditions. Specifically, the growth rates improved by up to 1.5‑fold for xylose (0.29 h^−1^) and 4.1‑fold for arabinose (0.60 h^−1^) (Fig. 2B, C), suggesting that adaptive mutations in the corresponding sugar catabolic pathways were acquired during evolution.

Beyond condition-specific improvements, notable unexpected impacts on alternative sugar catabolism were also observed. Notably, three GLC (1, 2, 4) and one ARA (1) isolates failed to grow on xylose, suggesting an inactivation of the heterologous xylose pathway (*vide infra*, Fig. 2A, C). Meanwhile, several GLC isolates exhibited enhanced growth on arabinose. Notably, the GLC1 and GLC4 strains showed elevated growth in arabinose medium, despite arabinose not being included in their original selection regime (Fig. 2A). All isolates evolved under simple selection pressures showed only marginal improvements in growth rate in mixed-sugar medium (4 g/L each of glucose, xylose, and arabinose), with a maximum increase of 1.1-fold relative to the JE3692 strain. These results indicate that sugar-specific adaptations may not translate into improved fitness in mixed or complex environments (Supplementary Fig. 2).

To identify genetic changes associated with the observed physiology, whole-genome sequencing was performed on the evolved isolates. These fitness improvements were observed on xylose and arabinose, whereas no mutations were identified in the glucose catabolic pathway of the GLC1-4 strains, consistent with no significant improvement in growth rate in glucose medium. Recurrent mutations were observed within each lineage: the XYL strains consistently carried mutations in only *xylB*, whereas the ARA strains shared mutations in *araD2*, *araC2*, *araE1*, or their intergenic region (Fig. 2D). Additionally, a native gene, PP_3603 encoding a transcriptional regulator of the glucuronate and glucarate catabolic pathways, was also mutated across ARA isolates, suggesting its involvement in arabinose catabolism.

We also identified endogenous genes that were repeatedly mutated across multiple evolved lineages, indicating convergent adaptation. Of the 28 mutations identified in regions repeatedly mutated across independent lineages, 12 mapped to functional genes, including PP_1650 (GacS, a two-component histidine kinase), PP_4171 (hypothetical protein), PP_4173 (two-component sensor histidine kinase/response regulator), and PP_4373 (FleQ, a transcriptional regulator) (Supplementary Fig. 3). We interpret these changes as generally beneficial adaptations to laboratory environments, based on their convergence across multiple ALE groups and observed occurrence in previous ALE studies with *P. putida* KT2440^41–43^.

The *xylB* gene emerged as a common target wherein mutations either improved or impaired cellular fitness on xylose (Supplementary Fig. 4). Mutations in *xylB* of the XYL1-4 strains occurred at amino acids 395 and 396 (Fig. 2D), which are highly conserved residues in XylB homologues (Supplementary Fig. 5). Disruptive *xylB* mutations included a frameshift (GLC4) and a premature stop codon (ARA1). The other isolates incapable of xylose catabolism exhibited an identical insertion mutation in the intergenic region between *xylE* and *xylA*, potentially reducing expression of the downstream xylose transporter and catabolic enzymes. Interestingly, these two divergent mutational strategies in the xylose pathway revealed the emergence of catabolic specialists and the trade-offs associated with a functional loss not under selection during the evolution experiments.

Interestingly, compared to the xylose pathway, no disruptive mutations were found in the arabinose pathway (Fig. 2D). This distinction is important because both pathways are heterologous, yet only one appears to be more favored. All 8 observed mutations in this pathway were found exclusively in ARA isolates: 3 occurred in the intergenic region upstream of the *araCDAB2* operon, and 5 were in the N-terminal region of the *araC2* or *araD2* genes, all of which were likely involved in regulating expression levels^44^. Strains with these mutations exhibited significantly increased growth rates in arabinose minimal media. Additionally, 3 mutations in the PP_3603 gene likely influence arabinose catabolism by modulating the expression of PP_3602, which shares the same functional annotation as the *araE2* gene (2-ketoglutarate semialdehyde dehydrogenase)^45,46^. Collectively, the phenotyping and genomic analysis of isolates evolved under simple selection pressures showed trends of condition-specific optimization and generation of catabolic specialists.

### Complex selection pressures promoted the emergence of catabolic generalists depending on experimental design

**Fig. 3:**
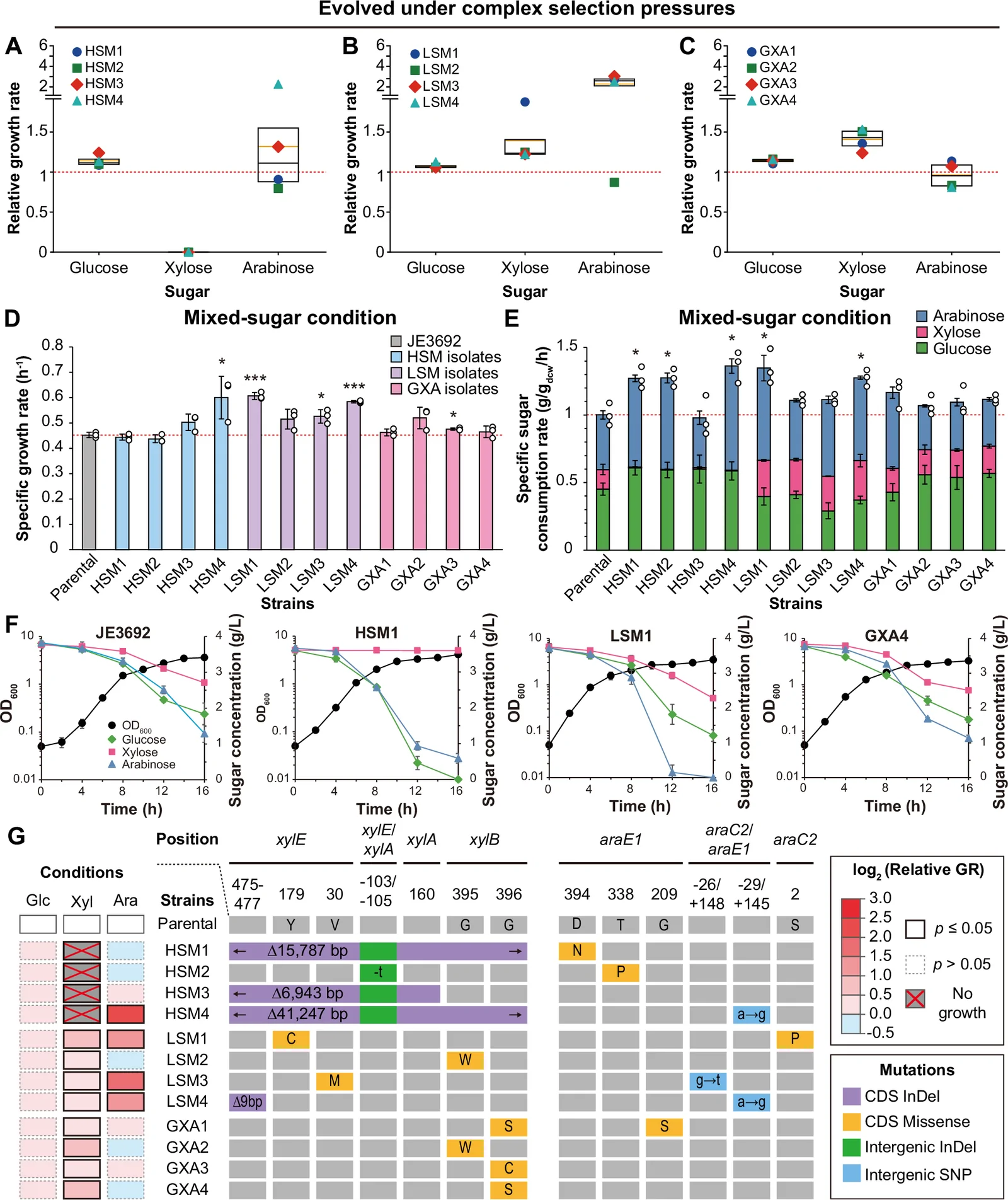
Complex selection pressures drive divergence between catabolic generalists and strains with trade-offs dependent on media conditions. Box plots show the relative growth rates of evolved strains isolated from **A** HSM, **B** LSM, and **C** GXA conditions under single-sugar conditions (glucose, xylose, and arabinose), compared to the parental strain, JE3692. Each dot represents the mean fold change in specific growth rate from duplicates (*n* = 2). Each box indicates the interquartile range, and each black and orange solid line in the box indicates the median and mean value, respectively. Red dotted lines indicate a fold change of 1. The *x*-axis represents sugar conditions under which the isolates were grown, and the *y*-axis represents the relative growth rate (fold change). **D** Specific growth rates of isolates evolved from complex selection pressures under the mixed-sugar conditions. The *x*- and *y*-axes indicate strains and specific growth rate (h^−1^), respectively. Red dotted lines indicate the specific growth rate of the JE3692 strain. **E** Specific sugar consumption rates of isolates evolved from complex selection pressures under the mixed-sugar conditions. The *x*- and *y*-axes indicate strains and sugar uptake rate (g/g_dcw_/h), respectively. Red dotted lines indicate the total specific sugar consumption rate of the JE3692 strain. **F** Growth and sugar consumption profiles of the parental strain (JE3692) and representative strains from each selection regime (HSM1, LSM1, and GXA4). The *x*-, left *y*-, and right *y*-axes indicate time (h), cell biomass (OD_600_), and sugar concentration (g/L), respectively. **G** Detailed mutational analysis related to xylose and arabinose catabolism in isolates evolved from complex selection pressures. All other details are the same as those described in Fig. 2D. For (**D**–**F**), bars or points represent mean values from three biological replicates (*n* = 3), and error bars indicate the standard deviations. White dots overlaid on the bar graphs in (**D**, **E**) represent individual biological replicates. Statistical significance was assessed using two-sided Student’s t-tests assuming equal variances. No adjustment was made for multiple comparisons. * and *** indicate *p* < 0.05 and *p* < 0.001, respectively. Bars without asterisks represent values that are not statistically significantly different from those of the JE3692 strain. Source data are provided as a Source Data file.

Evolution under complex selection pressures drove the divergence between catabolic specialists and generalists, depending on the specific selection criteria of each selection regime. As observed for the GLC and ARA isolates, the HSM isolates, which did not require complete sugar consumption for passage transfer, selectively lost the ability to grow on xylose as the sole carbon source (Fig. 3A), indicating that the xylose pathway is detrimental under these conditions. Two HSM isolates showed higher growth rates on arabinose alone whereas their growth rates on glucose alone remained similar to the unevolved parent. Conversely, the LSM and GXA isolates displayed enhanced growth rates on xylose (Fig. 3B, C). Evolution under the LSM condition also greatly enhanced the growth rate on arabinose, up to 3.0-fold, comparable to the growth rates of the ARA isolates. In contrast, the growth rates of the GXA isolates on arabinose alone were not significantly increased (Fig. 3C).

Growth and sugar consumption of isolates evolved under complex selection pressures were also evaluated under the mixed-sugar conditions containing 4 g/L each of glucose, xylose, and arabinose, identical to the HSM selection regime. Consistent with faster growth on individual sugars, the LSM isolates displayed generally higher growth rates than the other isolates (Fig. 3D). Among these, the LSM1 strain exhibited the highest growth rate (0.65 h^−1^), 1.4 times that of the JE3692 strain. Moreover, during the exponential growth phase, the LSM isolates generally exhibited higher specific sugar consumption rates than the other isolates (Fig. 3E). Among them, the LSM1 strain showed a total specific sugar consumption rate of 1.34 g/g_dcw_/h, which was 1.3-fold higher than that of the JE3692 strain. Its glucose consumption rate was slightly reduced, whereas the consumption rates for xylose and arabinose were 1.8- and 1.7-fold higher, respectively.

Time-course profiles of the parental strain (JE3692) and representative strains from each ALE group (HSM1, LSM1, and GXA4) revealed distinct sugar utilization patterns across regimes (Fig. 3F and Supplementary Fig. 6). The LSM1 strain exhibited enhanced utilization of all three sugars, with arabinose consumed most rapidly and nearly depleted within 16 h. In contrast, consistent with its inability to grow on xylose, the HSM1 strain did not consume xylose and instead rapidly depleted glucose and arabinose. While the GXA4 strain showed a modest improvement in xylose consumption relative to the parental strain, its overall utilization pattern remained largely similar to that of the parental strain.

Whole-genome sequencing of isolates evolved under complex selection pressures identified the mutations that occurred during evolution (Fig. 3G). First, a number of isolates carried mutations related to generally beneficial adaptation to laboratory environments, including mutations in PP_1650, PP_4171, PP_4173, and PP_4373, similar to those observed in isolates evolved under simple selection pressures (Supplementary Fig. 3). All the HSM isolates, which did not consume xylose, acquired large deletions in the *xylE*-*xylAB* region (HSM1, HSM3, HSM4) or an insertion upstream of *xylA* (HSM2), presumably leading to the observed elimination of xylose catabolism. In contrast, the LSM and GXA isolates, which retained the ability to utilize all three sugars, acquired non-disruptive mutations, defined here as sequence changes that did not result in loss-of-function, including substitutions or an in-frame deletion. In the arabinose pathway, mutations were identified in the protein sequences of *araE1* and *araC2*, as well as in the intergenic region upstream of *araC2*. However, mutations in *araE1* appeared to have no significant impact on fitness in arabinose medium (Fig. 3G). The exclusive occurrence of disruptive mutations in the xylose pathway, but not in the arabinose pathway, suggested distinct fitness constraints and motivated the hypothesis that the xylose isomerase pathway imposes a higher fitness cost (*vide infra*) (Supplementary Fig. 3).

### Evolutionary stability of heterologous sugar catabolism under divergent selection regimes

**Fig. 4:**
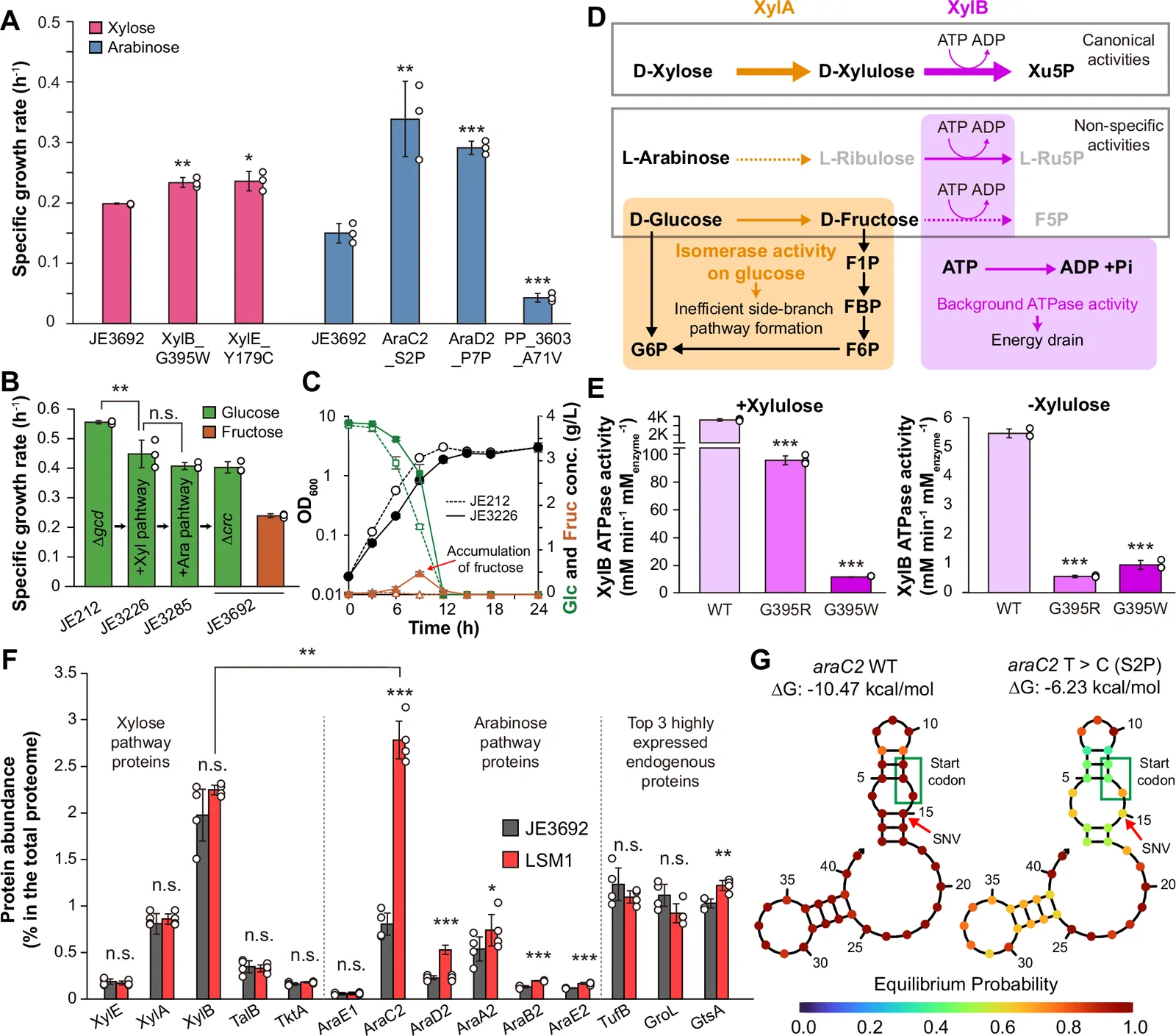
Evolution of heterologous sugar catabolism under divergent selective regimes. **A** Specific growth rates of JE3692 and reverse engineered strains cultured in xylose and arabinose minimal media. **B** Specific growth rates of the JE3692 and its intermediate strains cultured in glucose and fructose minimal media. The *x*- and *y*-axes represent strains and specific growth rate (h^−1^), respectively. **C** Detailed culture profiles of JE212 (dotted lines and open symbols) and JE3226 (solid lines and closed symbols) in glucose minimal media. The left and right *y*-axes indicate cell biomass (OD_600_) and concentration of glucose and fructose (g/L), respectively; the *x*-axis indicates time in hours (h). **D** Overview of the proposed evolutionary scenario underlying the negative selection of xylose pathway enzymes. Orange and purple boxes and arrows indicate enzymatic activities of XylA and XylB, respectively. Xu5P xylulose 5-phosphate, L-Ru5P L-ribulose 5-phosphate, F5P D-fructose 5-phosphate, G6P D-glucose 6-phosphate, F1P D-fructose 1-phosphate, FBP D-fructose 1,6-bisphosphate, F6P D-fructose 6-phosphate. **E** In vitro comparison of ATPase activity between wild-type and mutant XylBs in the presence and absence of D-xylulose. The *y*-axis represents ATPase activities (mM/min per mM enzyme), measured via NADH oxidation coupled to pyruvate kinase and lactate dehydrogenase reactions. **F** Comparison of the protein abundance levels of heterologous proteins for xylose and arabinose catabolism in JE3692 and LSM1. The *x*- and *y*-axes represent protein and the fraction in the total proteome, respectively. **G** Comparison of secondary structure of 5’ end of mRNA of the wild-type and mutant *araC2*, predicted by NUPACK^55^. The modified base and start codon are highlighted by red arrows and green boxes, respectively. For Fig. 4A–C, E, bars or points represent mean values from three biological replicates (*n* = 3). For Fig. 4F, bars represent mean values from four biological replicates (*n* = 4). Error bars indicate the standard deviations. Statistical significance was assessed using two-sided Student’s t-tests assuming equal variances. No adjustment was made for multiple comparisons. *, **, ***, and n.s. indicate *p* < 0.05, *p* < 0.01, *p* < 0.001, and *p* > 0.05, respectively. The white dots overlaid on the bar graphs represent data from individual replicates. Source data are provided as a Source Data file.

To assess the causal effects of key non-disruptive ALE-derived mutations, we reverse-engineered several genetic changes back into the parent JE3692 strain and assessed fitness changes under xylose and arabinose conditions. In the xylose pathway, we selected *xylB* G395W, the most convergent mutation in the xylose pathway, and *xylE* Y179C, identified in the LSM1 strain. In the arabinose pathway, we selected *araC2* S2P, identified in the LSM1 strain, *araD2* P7P, the most convergent mutation in the arabinose pathway, and PP_3603 A71V, one of the mutations observed in the ARA isolate. Except for PP_3603 A71V, these mutations independently increased growth rates under the corresponding conditions (Fig. 4A).

The PP_3603 A71V mutation exhibited a distinct phenotype compared to the other mutations tested. While it reduced the specific growth rate on arabinose (Fig. 4A), it eliminated the initial lag phase (Supplementary Fig. 7), suggesting a trade-off between lag duration and maximal growth. Notably, PP_3603 mutations were observed exclusively in the ARA isolates and co-occurred with mutations in *araC2* or *araD2* (Fig. 2D), suggesting that their combined effects may contribute to improved fitness under arabinose conditions. These observations suggest that PP_3603 may play a role in modulating condition-specific adaptation rather than directly enhancing growth rate, although further mechanistic investigation is required.

In a separate assay, to understand the reason for the selective disruption of the xylose pathway during ALE, we examined whether the expression of the xylose and arabinose pathways imposed fitness costs under non-selective regimes using phenotypic assays. To this end, we cultivated the JE3692 and its intermediate strains in glucose minimal medium (Fig. 4B). The strains utilized were JE212, which lacks both the xylose and arabinose pathways; JE3226, which contains only the xylose pathway; and JE3285, which contains both pathways. All three intermediate strains possessed an intact *crc* gene, which was deleted in the JE3692 strain. The JE3226 strain exhibited a 19% lower growth rate (0.45 ± 0.01 h^−1^, *p* < 0.01) compared to the JE212 strain (0.56 ± 0.05 h^−1^). Although the JE3285 strain showed a slightly lower growth rate (0.41 ± 0.01 h^−1^) than the JE3226 strain (a 9% decrease), the difference was not statistically significant (*p* > 0.05). We also observed a transient accumulation of up to 0.5 g/L fructose in the JE3226 culture grown in glucose minimal medium, whereas only negligible levels were detected in the JE212 culture (Fig. 4C). Taken together, these results demonstrated that the xylose pathway reduced growth fitness under non-selective conditions, consistent with its selective loss during ALE in the absence of direct selection pressures.

To determine whether non-specific enzymatic activities of the xylose catabolic enzymes contributed to fitness costs and pathway instability during ALE^19,47–49^, we examined the biochemical activities of core enzymes, XylA and XylB, using in vitro enzyme assays (Fig. 4D). In the XYL, LSM, and GXA groups, at least one isolate from each carried a substitution at residue 395 of XylB, and thus we compared the activities of the mutant XylB variants (G395R and G395W) (Figs. 2D and 3G). In in vitro enzyme assays, wild-type XylB exhibited high activity on D-xylulose and even retained background ATPase activity in the absence of a substrate (Fig. 4E), consistent with previous studies^19,48^. Interestingly, the G395R and G395W variants exhibited markedly reduced xylulose activity, by 37-fold and 330-fold, respectively, despite having evolved under xylose-selective conditions. They also decreased background ATPase activity by 9.9‑ and 5.7‑fold, respectively. Analysis of the AlphaFold-predicted structure suggested that residues 395 and 396 are positioned adjacent to the ATP-binding region (Supplementary Fig. 8), indicating that these substitutions may alter ATP binding affinity^50^. Although the background ATPase activity of wild-type XylB was only about 0.15% of that observed with D-xylulose, its high expression level may increase ATP turnover in the absence of xylose, potentially contributing to the impaired growth (Fig. 4B). This outcome suggested that maximizing enzyme activity was not the primary determinant of fitness under selection. Accordingly, under strong selection pressures for xylose utilization (XYL, LSM, and GXA), XylB activity appeared to be fine-tuned while ATPase activity was reduced. In contrast, under weaker xylose selection pressures (GLC, ARA, and HSM), evolution tended to disrupt XylB or the entire xylose pathway.

The non-specific isomerase activity of XylA toward D-glucose may also influence fitness in this context. Proton NMR analysis qualitatively confirmed that XylA exhibited isomerase activity toward all three target sugars; high for D-xylose, moderate for D-glucose, and minimal for L-arabinose (Supplementary Fig. 9)^47,49^. This activity toward glucose explains the transient accumulation of fructose in the JE3226 culture (Fig. 4C). The formation of an inefficient side branch via unnecessary D-fructose production, accompanied by flux imbalance and diauxic sugar utilization^51,52^, may have imposed selective pressures to eliminate XylA during ALE experiments^53,54^. Further support comes from the observation that the JE3692 strain showed a 1.9-fold lower growth rate when grown on D-fructose than on D-glucose, likely due to the absence of phosphofructokinase (Fig. 4B). This may explain why, in ALE regimes with strong selection pressures on glucose catabolism (GLC and HSM), not only XylB but the entire xylose pathway was disrupted by large deletions or mutations in intergenic regions.

In another drill-down assay on ALE-derived findings, proteomic analysis suggested that the high protein abundance of XylA and XylB may exacerbate the fitness burden imposed by the xylose isomerase pathway. XylB accounted for 2% and XylA for 0.8% of the total protein by mass (Fig. 4F). Notably, the protein abundance of XylB was comparable to the total abundance of arabinose catabolic enzymes and even exceeded that of the three most highly expressed endogenous proteins: TufB (elongation factor Tu-B), GroL (chaperonin), and GtsA (mannose/glucose ABC transporter substrate-binding protein) (Supplementary Data 2). These quantitative proteomic patterns indicate that high protein abundance of XylA and XylB amplified their non-specific activities and, in turn, the likely burdensome effects.

Finally, to investigate the arabinose pathway, we focused on the AraC2 S2P mutation and its associated proteomic changes in the LSM1 strain. Notably, comparative proteomic analysis revealed that AraC2 abundance in the LSM1 strain was 3.5-fold higher than in the JE3692 strain, due to the S2P mutation (Fig. 4F). Furthermore, downstream arabinose catabolic enzymes, including AraD2, AraA2, AraB2, and AraE2, also showed increased protein abundance. These increases were likely attributable to a mutation near the 5′ end of *araC2* that destabilized the mRNA secondary structure around the ribosome-binding site, thereby improving ribosome accessibility and translation efficiency (Fig. 4G)^55^. Indeed, based on UTR Designer predictions^44^, the mutant *araC2* was expected to show a 3.4-fold increase in expression.

Collectively, these findings demonstrated that evolutionary stability of heterologous pathways is determined by the balance between selection pressure and pathway-specific fitness costs. The arabinose pathway, which imposed minimal fitness burden, was stably retained across conditions. In contrast, the xylose pathway incurred substantial fitness costs and was therefore maintained only under regimes with strong selection for xylose utilization, where evolution favored mutations that reduced these costs rather than maximizing catalytic activity.

### An evolved isolate with a generalist sugar-catabolism phenotype enhanced indigoidine production

**Fig. 5:**
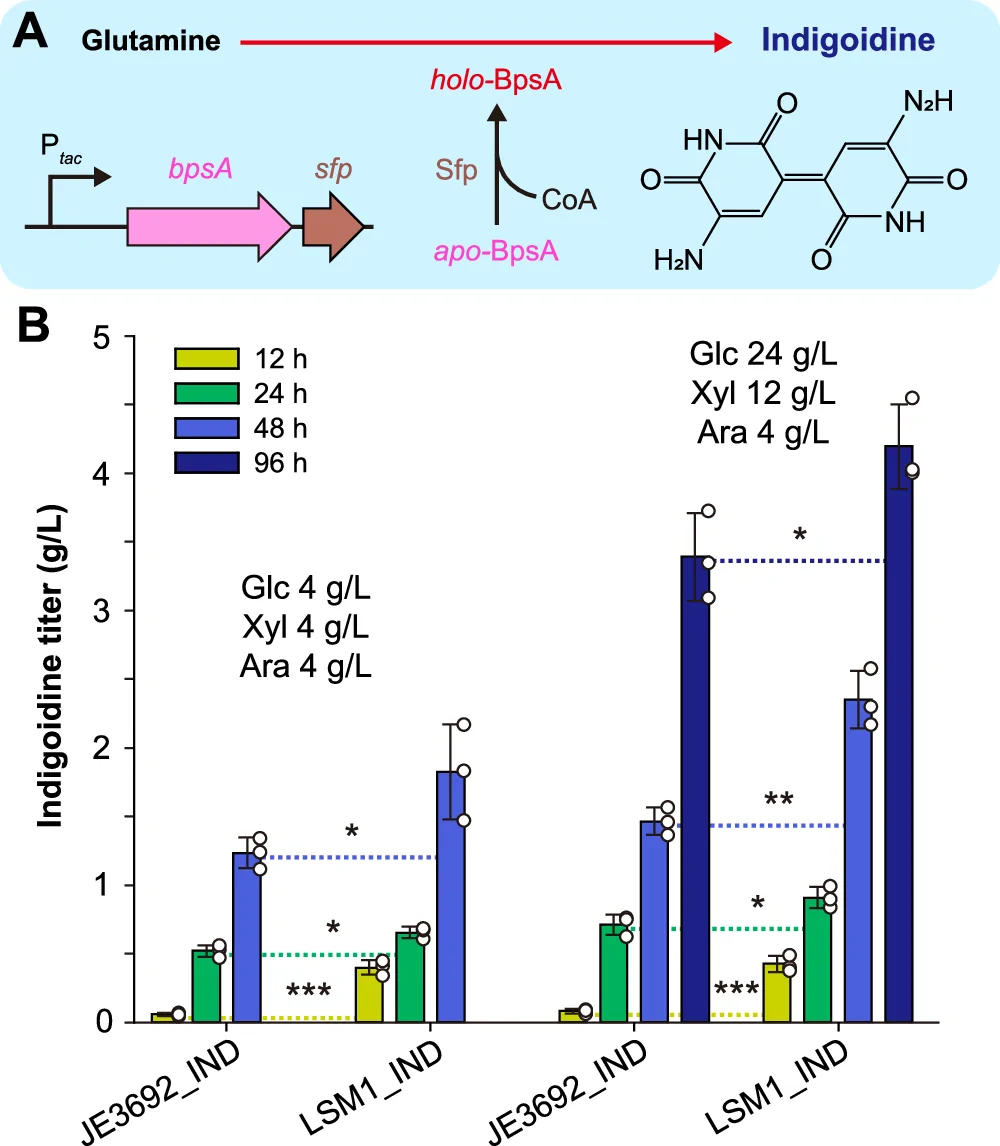
Indigoidine production from lignocellulosic sugars using an evolved strain as a host. **A** The indigoidine production pathway in engineered *P. putida* strains. Indigoidine can be produced from glutamine by heterologous expression of *bpsA* from *S. lavendulae* and *sfp* from *B. subtilis*. These genes were expressed under the Tac promoter (P_tac_) and the cassette was introduced into a plasmid. **B** Comparison of the indigoidine titers (g/L) of engineered strains based on the parental strain (JE3692) and an evolved strain (LSM1) after 12 h, 24 h, 48 h, and 96 h cultivation under isotonic sugar conditions (4 g/L of each sugar) and mock hydrolysate conditions (24 g/L of glucose, 12 g/L of xylose, and 4 g/L of arabinose). The *x*- and *y*-axes indicate producer strain and indigoidine titer (g/L). Bars represent mean values from three biological replicates (*n* = 3), and error bars indicate the standard deviations. Statistical significance was assessed using two-sided Student’s t-tests assuming equal variances. No adjustment was made for multiple comparisons. *, **, and *** indicate statistical significance (*p* < 0.05, *p* < 0.01 and *p* < 0.001, respectively) between cultures of two different strains measured at the same timepoint. The white dots overlaid on the bar graphs represent data from individual replicates. Source data are provided as a Source Data file.

The potential of the evolutionarily optimized LSM1 strain as a production host for conversion of lignocellulosic sugars was evaluated using indigoidine production, a natural blue pigment with various industrial applications, as a relevant bioproduct. To achieve this, we introduced the biosynthetic pathway for indigoidine into both the parental strain and the LSM1 strain (Fig. 5A and Supplementary Table 3)^56–59^. This heterologous pathway included the *bpsA* gene encoding blue pigment synthetase A from *Streptomyces lavendulae* and the *sfp* gene encoding 4′-phosphopantetheinyl transferase from *Bacillus subtilis*^60,61^. Using the plasmid system, these two genes were expressed under the *tac* promoter without *lacI*, which has previously been used for strong and constitutive expression in *P. putida* KT2440^62,63^. The resulting plasmid, pTE252_IND, was transformed into JE3692 and LSM1 to generate the JE3692_IND and LSM1_IND strains. These strains were cultivated in isotonic sugar media containing 4 g/L each of glucose, xylose, and arabinose, as well as under mock hydrolysate conditions, which contained 24 g/L of glucose, 12 g/L of xylose, and 4 g/L of arabinose^64^.

The LSM1_IND strain demonstrated a consistently higher indigoidine titer compared to the JE3692_IND strain (Fig. 5B). Under isotonic conditions, the LSM1_IND strain produced 1.8 g/L of indigoidine over 48 h, 1.5-fold higher compared to the JE3692_IND strain under the same conditions. When grown on mock hydrolysate, the LSM1_IND strain achieved a titer of 4.2 g/L over 96 h, 1.2-fold higher compared to the JE3692_IND strain. This titer improvement is consistent with optimized xylose and arabinose catabolism and metabolic flux redistribution described in the previous sections. The difference in titer between the two strains was more pronounced in the early stages of cultivation, with the LSM1_IND strain exhibiting 6.9-fold and 5.2-fold higher titers than the JE3692_IND strain after 12 h under isotonic and mock hydrolysate conditions, respectively. These results demonstrate the utility of ALE for improving the conversion of mixed substrates into a single value-added product.

## Discussion

In this study, we conducted an automated ALE campaign on a previously engineered *P. putida* KT2440 strain, JE3692^23^, under various simple and complex selection pressures to optimize lignocellulosic sugar catabolism. Isolates evolved under simple selection pressures generally exhibited improved growth relative to the starting strain only under their respective selection conditions. Among isolates evolved under complex selection pressures, those from the HSM and GXA regimes likewise evolved into catabolic specialists. In contrast, LSM isolates, evolved under mixed-sugar conditions that required complete consumption of all three sugars, functioned as catabolic generalists with robust growth across single- and mixed-sugar environments (Fig. 3B, D, E). These findings indicate that LSM is an effective selection regime for the simultaneous optimization of multi-substrate catabolism within a single ALE experiment. Furthermore, the ALE-derived LSM1 strain led to improved production of indigoidine, demonstrating utility for biomanufacturing goals (Fig. 5B).

The distinct mutational landscapes and variations in catabolic performance across evolved isolates from complex selection pressure regimes provided valuable insights into the rational design of evolutionary engineering strategies. The GXA regime, despite imposing explicit temporal variation in substrate availability, did not effectively yield robust generalists. Relatively rapid alternating selection pressures across passages likely delayed the fixation of sugar-specific beneficial mutations and limited the accumulation of adaptive mutations across multiple pathways within the same lineage^65^. Instead, rapid substrate switching may have favored population-level specialization, in which distinct subpopulations adapted to individual sugars rather than converging on a generalist phenotype. Nevertheless, the GXA1 strain showed phenotypic and genotypic features similar to the LSM isolates, suggesting that sequential substrate exposure can still converge toward generalist-like solutions. This observation raises the possibility that a longer overall ALE experiment duration or increasing the interval between substrate shifts may promote the emergence of more robust generalist phenotypes.

The HSM regime favored the emergence of catabolic specialists due to nutrient-rich conditions that prioritized rapid utilization of preferred substrates (Fig. 3E, F). This outcome likely reflects differences in sugar concentrations and transfer criteria during the ALE experiments. Under the HSM selection regime, carbon remained abundant until transfer, likely favoring early sweeps of sprinters that rapidly dominated the population by utilizing easily catabolizable carbon sources^66^. However, such dynamics might not enforce sustained selection for a high overall growth rate across the desired time frame for applications. Consequently, HSM isolates often lost xylose catabolic ability (which likely imposed fitness costs) and evolved as specialists on glucose and arabinose. These results provide a mechanistic example of how fitness costs associated with specific catabolic pathways may contribute to hierarchical sugar utilization in nutrient-rich and stable environments^10,11^.

By contrast, the LSM regime maintained low carbon availability and required complete sugar depletion before transfer, imposing selection for efficient use of all three sugars and promoting generalists^66^. Furthermore, disruptive mutations in flagellar biosynthetic genes (*fliR* and *flgH*) and associated regulatory genes (*fliA* and PP_5263 annotated as a GGDEF domain protein) in LSM isolates (Supplementary Data 1) are likely attributable to more efficient proteome reallocation and reduced motility or biofilm formation that increase specific growth rate^67,68^. Together, these regime-dependent outcomes emphasize the need to rationally design ALE conditions that preserve and co-optimize targeted metabolic pathways while accounting for evolutionary parameters such as mutation rates and population sizes^39^.

However, because our analyses were based on selected clonal isolates, population-level adaptive solutions under complex selection pressures may not have been fully captured. Although the clone isolation strategy successfully yielded isolates with improved mixed-sugar utilization compared to the starting strain, a limitation of this study is that we did not use high-throughput screening to thoroughly explore evolved population diversity. Consequently, better-performing or more representative generalist clones may have remained unsampled within the evolved populations. Combining ALE with high-throughput automated screening represents a promising technological advancement for future studies. Furthermore, periodic deep population sequencing could reveal whether stable polymorphisms or less abundant specialist lineages contributed to overall population performance^69,70^.

The utility of the generalist phenotype was demonstrated by enhanced indigoidine production, which was driven by the coordinated utilization of multiple substrates rather than reliance on a single carbon source. Notably, the LSM1_IND strain achieved higher titer under mock hydrolysate conditions (24 g/L glucose, 12 g/L xylose, and 4 g/L arabinose) than under isotonic conditions (4 g/L each sugar) (Fig. 5B). This increase occurred despite identical arabinose concentrations, suggesting that arabinose contributed to 2-ketoglutarate generation, while the additional energy and carbon supplied via the xylose isomerase pathway further enhanced production. The pathway design, featuring complementary metabolic entry points and their balanced, coordinated optimization, likely enhanced production by supporting both precursor generation and the overall metabolic capacity required for higher output.

Our results highlighted the metabolic trade-offs associated with the xylose isomerase pathway, consistent with previous reports^71–73^. In this study, optimization of this pathway was constrained by intrinsic fitness costs arising from non-specific enzyme activities, which shaped evolutionary outcomes across selection regimes. More broadly, these findings indicate that when heterologous pathways impose a severe fitness burden, ALE may preferentially select for mutations that reduce this cost rather than mutations that directly improve enzyme kinetics. While the LSM strategy successfully forced the retention and coordination of these pathways rather than their complete loss, further improvements to combined sugar utilization may require complementary ALE strategies. For example, implementation of alternative oxidative routes, such as the Weimberg pathway^24,26^, may enhance robustness by alleviating these trade-offs. In parallel, burden-relieved strains such as LSM1 could serve as starting points for targeted rational engineering or subsequent ALE campaigns, potentially under more complex alternating selection regimes or continuous culture setups, to shift evolutionary pressure away from basic burden reduction and toward mutations that enhance catalytic capacity and combined sugar uptake.

While the LSM regime enabled the evolution of generalist phenotypes, industrial translation of this phenotype will require both further strain validation and appropriate process configuration. Industrial bioprocesses often operate at higher substrate loads, which can impose physiological burdens, as reflected by the reduced growth rates of both JE3692 and LSM1 at elevated sugar concentrations (Supplementary Fig. 10). Nevertheless, LSM1 outperformed the parental strain under these conditions in both growth rate (Supplementary Fig. 10) and production (Fig. 5B), indicating that the generalist phenotype provides a robust metabolic foundation beyond its original selection regime. However, its performance under industrially relevant stresses, including pretreatment-derived inhibitors and product-associated burdens, remains to be determined^74,75^. At the process level, substrate-limited strategies that minimize free-sugar accumulation, such as analytically guided fed-batch operation, may help preserve the generalist phenotype by limiting prolonged selection for catabolic specialists. Alternatively, evolutionary engineering campaigns focused on, for example, osmolarity tolerance with LSM1 as a starting strain or combined into a complex alternating selection regime can be applied when confounding industry-scale stresses are well defined^76^.

This study represents a highly complex and parallelized ALE investigation, enabled by the use of automated robotic ALEbot platforms^39,77,78^. Prior plate-based automated ALE studies typically relied on fixed schedules, such as transferring once per day or upon all cultures reaching a defined state, which limited the implementation of diverse selection regimes, particularly under regimes such as LSM and HSM^79–81^. In this study, each lineage was cultivated in a separate tube, enabling individual OD tracking and timely transfers that maintained consistent selective regimes and promoted distinct evolutionary trajectories. Our results demonstrate that the complex selection pressure regime that enforced complete consumption of all sugars can optimize multi-substrate catabolism, highlighting its advantage over traditional ALE that relies on a single selection pressure. Looking ahead, we expect that this ALE strategy based on complex selection pressures could be systematically applied to even more complex and heterogeneous feedstocks, such as lignin-derived aromatics, marine polysaccharides, and depolymerized mixed plastic waste^43,82–84^.

## Methods

### Bacterial strains, plasmids, and reagents

Strains and plasmids used in this study are listed in Supplementary Table 3. Plasmids were primarily constructed using the NEBuilder HiFi DNA Assembly Kit (New England Biolabs, Ipswich, MA, USA). Oligonucleotides, synthesized by Integrated DNA Technologies (IDT, Coralville, IA, USA), are listed in Supplementary Table 4. The plasmids used for recombination were based on the pK18mobsacB backbone (ATCC 87097) and recombination regions either cloned from isolates ARA3, LSM1, LSM2 or synthesized by GenScript (Piscataway, NJ, USA). For DNA amplification, the Q5 High-Fidelity DNA polymerase or OneTaq DNA polymerase (NEB) was used. Plasmids were purified using a Zymo PURE Plasmid Miniprep kit from Zymo Research (Irvine, CA, USA). Genomic DNA samples were prepared by using a Quick-DNA Fungal/Bacterial Miniprep kit from Zymo Research. Genome-sequencing library samples were prepared using an NEBNext Ultra II kit from NEB (Ipswich, MA, USA). All chemicals were purchased from Sigma-Aldrich (St. Louis, MO, USA) and used without further purification.

### Culture conditions

Bacterial cultivations were conducted in a Luria−Bertani (LB, 10 g/L tryptone, 5 g/L yeast extract, 10 g/L NaCl) or modified minimal M9 medium (4 g/L glucose, 2 g/L (NH_4_)_2_SO_4_, 6.8 g/L Na_2_HPO_4_, 3 g/L KH_2_PO_4_, 0.5 g/L NaCl, 2 mM MgSO_4_, 0.1 mM CaCl_2_, 500 μL/L 2000× trace element solution). The composition of the trace element solution is 4.5 g/L ZnSO_4_·7H_2_O, 0.7 g/L MnCl_2_·4H_2_O, 0.3 g/L CoCl_2_·6H_2_O, 0.2 g/L CuSO_4_·2H_2_O, 0.4 g/L Na_2_MoO_4_·2H_2_O, 4.5 g/L CaCl_2_·2H_2_O, 3.0 g/L FeSO_4_·7H_2_O, 1.0 g/L H_3_BO_3_, 0.1 g/L KI, 15 g/L disodium ethylenediaminetetraacetate^42,85^. For production strains only, gentamicin was supplemented at 30 μg/mL to maintain the plasmid carrying the indigoidine biosynthetic genes. Cultures were performed in 35 mL cylindrical tubes (Roche, Basel, Switzerland; order no. 58.53) containing 17 mL of medium at 30 °C, agitated at 1100 rpm with a magnetic stirring bar.

### Adaptive laboratory evolution

For ALE, cultures were passaged by transferring 150 μL into 17 mL of fresh medium using an automated robotic platform^39,77^, corresponding to an ~1:110 dilution. In the LSM selection regime, transfers were performed at OD_600_ of 0.40, corresponding to complete depletion of all sugars and an effective bottleneck size of ~6 × 10^7^ cells, assuming that an OD_600_ of 1 corresponds to ~1 × 10^9^ cells/mL. In the other selection regimes, transfers were performed at OD_600_ of 0.55, corresponding to an effective bottleneck size of ~8.3 × 10^7^ cells. OD_600_ was measured using a Sunrise^TM^ microplate reader from Tecan (Männedorf, Switzerland).

### Clonal isolation

Clonal isolates were obtained from the endpoint populations by streaking cells onto agar plates containing the respective selection sugars; however, for the GXA condition, cells were plated on xylose, the primary target for improvement. Three randomly selected fast-growing colonies were inoculated into deep-well plates (200 μL culture volume) and cultivated under the corresponding conditions. The isolate demonstrating the highest overall sugar consumption was selected as the representative clone for further analysis.

### Whole-genome sequencing

Raw sequencing reads were obtained by using a NovaSeq 6000 (Illumina) at the UC San Diego IGM Genomics Center and analyzed by using Breseq (version 0.35.4)^86^ and Bowtie2 (version 2.2.6)^87^. Mutations consistently present in the starting strain were filtered out (Supplementary Table 5). The summarized analysis results are also included in Supplementary Data 1.

### Protein expression and purification

Genes encoding XylA and XylB were cloned into a pET15b vector and transformed into *E. coli* BL21(DE3). Cultures were grown at 37 °C in LB medium containing 50 µg/mL ampicillin until reaching an OD_600_ of 0.6–0.8, at which point expression was induced with 1 mM IPTG and continued overnight at 18 °C. Cells were harvested by centrifugation at 4000 × *g* for 30 min at 4 °C, resuspended in lysis buffer (20 mM TRIS pH 8, 300 mM NaCl, 5 mM imidazole) and lysed by sonication on ice. Lysates were clarified by centrifugation and purified using nickel affinity chromatography (Ni-NTA IMAC). Eluted fractions were dialyzed (20 mM TRIS pH 8, 300 mM NaCl), concentrated to >10 mg/mL, and flash frozen dropwise in liquid nitrogen, then stored at −80 °C until use. XylA concentration was determined by absorbance at 280 nm using a calculated extinction coefficient of 71,070 M^−1^cm^−1^, and XylB concentration was determined by absorbance at 280 nm using a calculated extinction coefficient of 82,756 M^−1^cm^−1^.

### NMR analysis of XylA activity

Reactions were performed in 100 mM potassium phosphate buffer (pH 8) containing 10 mM MgSO_4_, 20 mM sugar substrate (D-xylose, D-glucose, or L-arabinose), and 12 µM purified XylA. Reaction mixtures (final volume: 600 µL) were supplemented to reach 10% D_2_O and 0.1 mg/mL TMSP (trimethylsilyl propionic acid) as an internal chemical shift and integration reference. ^1^H NMR spectra were acquired on a 400 MHz NMR spectrometer at room temperature using a standard one-dimensional water-suppression pulse sequence. Spectra were processed and analyzed in Mestrenova (Mestrelab Research). Product peaks for D-xylulose and D-fructose were identified based on available published or reference spectra and quantified by peak integration. The L-ribulose product lacked published assignments; thus, quantification was based on a representative signal assumed to correspond to one or more protons (≥1 H). Final concentrations were estimated using relative integration values calibrated to the TMSP internal standard.

### Coupled enzymatic assay to detect XylB activity

XylB activity was monitored using a continuous, coupled assay with pyruvate kinase (PK) and lactate dehydrogenase (LDH) linking ATP consumption to NADH oxidation. Reactions were conducted in 200 mM potassium phosphate buffer (pH 8.0) supplemented with 20 mM MgSO_4_, 2 mM phosphoenolpyruvate (PEP), 0.01X PK/LDH enzyme mix (with the 1X mixture corresponding to ~600–1000 U/mL pyruvate kinase and ~900–1400 U/mL LDH), 1 mM NADH, and 0.04 µM purified XylB. Sugar substrates were added at 10 mM final concentration, except D-xylulose, which was added at 2 mM. The final reaction volume was 200 µL and reactions were performed in 96-well UV-STAR microplates (Greiner Bio-One). Absorbance at 340 nm (A_340_) was monitored at 1-minute intervals for 60 min at room temperature using a BioTek Synergy H1 plate reader.

### Proteome analysis

*P. putida* cultures were collected after the OD_600_ reached 0.6 in minimal media supplemented with 4 g/L of glucose. Cells were harvested and stored at −80 °C until further processing. Protein was extracted from cell pellets and tryptic peptides were prepared following the established proteomic sample preparation protocol^88^. Briefly, cell pellets were resuspended in Qiagen P2 Lysis Buffer (Qiagen, Germany) to promote cell lysis. Proteins were precipitated with the addition of 1 mM NaCl and 4× vol acetone, followed by two additional washes with 80% acetone in water. The recovered protein pellet was homogenized by pipetting with 100 mM ammonium bicarbonate in 20% methanol. Protein concentration was determined by the DC protein assay (BioRad, USA). Protein reduction was accomplished using 5 mM tris 2-(carboxyethyl)phosphine (TCEP) for 30 min at room temperature, and alkylation was performed with 10 mM iodoacetamide (IAM; final concentration) for 30 min at room temperature in the dark. Overnight digestion with trypsin was accomplished with a 1:50 trypsin:total protein ratio.

The resulting peptide samples were analyzed on an Agilent 1290 UHPLC system coupled to a Thermo Scientific Orbitrap Exploris 480 mass spectrometer for discovery proteomics^89^. Briefly, peptide samples were loaded onto an Ascentis® ES-C18 Column (Sigma–Aldrich, USA) and were eluted from the column by using a 10-min gradient from 98% solvent A (0.1 % FA in H2O) and 2% solvent B (0.1% FA in ACN) to 65% solvent A and 35% solvent B. Eluting peptides were introduced to the mass spectrometer operating in positive-ion mode and were measured in data-independent acquisition (DIA) mode with a duty cycle of 3 survey scans from m/z 380 to m/z 985 and 45 MS2 scans with precursor isolation width of 13.5 m/z to cover the mass range.

DIA raw data files were analyzed by the integrated software suite DIA-NN^90^. The databases used in the DIA-NN search (library-free mode) are the latest *P. putida* Uniprot proteome FASTA sequences plus the protein sequences of the heterologous proteins and common proteomic contaminants. DIA-NN determines mass tolerances automatically based on first pass analysis of the samples with automated determination of optimal mass accuracies. The retention time extraction window was determined individually for all MS runs analyzed via the automated optimization procedure implemented in DIA-NN. Protein inference was enabled, and the quantification strategy was set to Robust LC = High Accuracy. Output main DIA-NN reports were filtered with a global FDR = 0.01 on both the precursor level and protein group level. The Top3 method, which is the average MS signal response of the three most intense tryptic peptides of each identified protein, was used to plot the quantity of the targeted proteins in the samples^91,92^. The processed data and analysis results are provided in Supplementary Data 2.

### Metabolite quantification

Indigoidine was quantified after its extraction using dimethyl sulfoxide as described previously^56^. For indigoidine extraction, 1 mL of culture was centrifuged at 21,000 × *g* for 3 min, and the supernatant was discarded. The cell pellet was resuspended with 100 μL of acid-washed beads and 1 mL of DMSO containing 2% Tween® 20, followed by two 1-min vortexing cycles. After centrifugation at 21,000 × *g* for 3 min, the indigoidine concentration was determined by measuring the OD612 of the supernatant. OD_612_ was measured by using an Infinite^®^ 200 PRO microplate reader from Tecan (Männedorf, Switzerland), and the concentration was determined from a calibration curve generated with indigoidine standards of known concentrations (Supplementary Fig. 11). Concentrations of sugars were quantified by using a 1260 Infinity II LC system (Agilent, Santa Clara, CA, USA) equipped with an HPX-87H column (Biorad, Hercules, CA, USA) and a refractive index detector. 5 mM H_2_SO_4_ was used as the mobile phase at a flow rate of 0.5 mL/min. The column temperature was maintained at 60 °C.

### Quantification of growth and substrate utilization

The specific growth rate (μ, h^−1^) was determined from the linear regression of the natural logarithm of biomass (OD_600_) versus time (h) during the exponential growth phase (~8 h). Biomass concentration (g_dcw_/L) was estimated from OD_600_ using a conversion factor of 0.38 g_dcw_/L per unit OD_600_. Specific sugar consumption rates (*q*_*s*_, g/g_dcw_/h) were calculated over the exponential growth phase as the amount of substrate consumed (g/L) divided by the biomass-time integral. The biomass-time integral was estimated assuming exponential growth, using the measured specific growth rate (μ) to describe biomass accumulation over the selected interval. For total sugar consumption rates, rates were calculated for each sugar individually and then summed.

### Statistics and reproducibility

No statistical method was used to predetermine sample size. Sample sizes were chosen based on standard practice for microbial phenotyping experiments and are indicated in the figure legends and Source Data file. Biological replicates represent independently grown cultures or independently isolated evolved strains, as specified in the figure legends. No data was excluded from the analysis. The experiments were not randomized because the microbial strains and culture conditions were predefined by the experimental design and processed under the same laboratory conditions. The investigators were not blinded to allocation during experiments and outcome assessment because the measurements were based on objective quantitative readouts. Statistical analyses were performed using two-sided Student’s t-tests assuming equal variances. No adjustment was made for multiple comparisons.

### Reporting summary

Further information on research design is available in the Nature Portfolio Reporting Summary linked to this article.

## Supplementary information


Supplementary Information
Description of Additional Supplementary Files
Supplementary Data 1
Supplementary Data 2
Reporting Summary
Transparent Peer Review file


## Source data


Source data


## Data Availability

The raw genome-sequencing files are available at the National Center for Biotechnology Information (NCBI) Sequence Read Archive (SRA) under accession code PRJNA1395279 (Supplementary Table 6) [https://www.ncbi.nlm.nih.gov/bioproject/PRJNA1395279/]. The variant-calling data is available in ALEdb v1.0^93^ under project name Pputida_complex_ALE [https://aledb.org/ale/project/259/]. The generated mass spectrometry proteomics data have been deposited in the ProteomeXchange Consortium via the PRIDE^94^ partner repository with the dataset identifier PXD055215. Protein structures used in this work include PDB IDs 2ITM and 3LL3. The *P. putida* KT2440 UniProt proteome FASTA used for DIA-NN searches was obtained from UniProt proteome UP000000556. DIA-NN and AlphaFold 3 are freely available online [https://github.com/vdemichev/DiaNN; https://github.com/google-deepmind/alphafold3]. All data generated in this study are provided in the Source Data file. Source data are provided with this paper.
