# Supplementary material for: Simultaneous optimization of lignocellulosic sugar catabolism via systematic laboratory evolution under complex selection pressure
Source: Nat Commun. 2026 Jul 29;17:9194. doi: 10.1038/s41467-026-75974-x (PMC13522501; doi:10.1038/s41467-026-75974-x)
Supplement: Supplementary file 1 [file 41467_2026_75974_MOESM1_ESM.pdf]

Supplementary Information of

Simultaneous optimization of lignocellulosic sugar catabolism via systematic laboratory evolution under complex selection pressure

Sunghwa Woo[a,b,1], Hyun Gyu Lim[a,b,c,d,1], Brenna Norton-Baker[e,f], Torrey M. Lind[e,f], Nathan E. Gladden[f,g], Yan Chen[b,h], Ciara de Venecia[a], Thomas Eng[b,h], Christopher W. Johnson[e,f], Aindrila Mukhopadhyay[b,h], Christopher J. Petzold[b,h], Adam M. Guss[f,g], Gregg T. Beckham[e,f], Adam M. Feist[a,b,i,*]

[a]Department of Bioengineering, University of California San Diego, 9500 Gilman Dr., La Jolla, CA 92093, USA

[b]Joint BioEnergy Institute, 5885 Hollis Street, 4th floor, Emeryville, CA 94608, USA

[c]Department of Biological Sciences and Bioengineering, Inha University, Inha-ro 100, Michuhol-gu, Incheon 22212, Korea

[d]Institute of AI-driven Industrial Biotechnology, Inha University, Incheon 22212, Korea

[e]Renewable Resources and Enabling Sciences Center, National Laboratory of the Rockies, Golden, CO 80401, USA

[f]Agile BioFoundry, Emeryville, CA 94608, USA

[g]Biosciences Division, Oak Ridge National Laboratory, Oak Ridge, Tennessee, United States of America

[h]Biological Systems and Engineering Division, Lawrence Berkeley National Laboratory, Berkeley, CA 94720, USA

[i]BRIGHT, Technical University of Denmark, 2800 Kgs., Lyngby, Denmark

[1]These authors made equal contributions to this work. [*]Corresponding author, afeist@ucsd.edu

Contents

Number of pages: 21

Number of figures: 11

Number of tables: 6

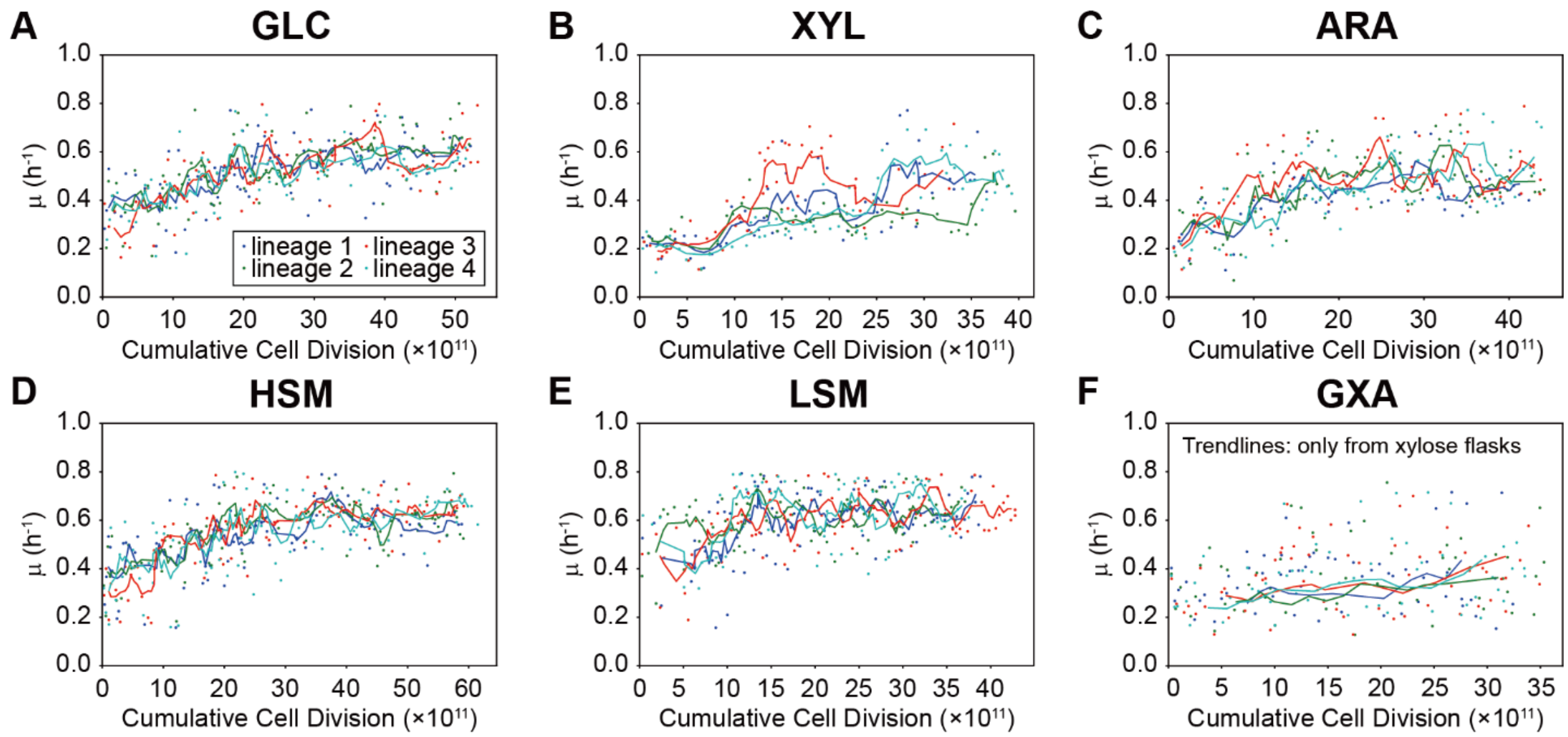

Supplementary Figure 1. ALE trajectories of lineages evolved under simple and complex selection pressures

Growth trajectories during the ALE under simple selection pressure ((A) GLC, (B) XYL, (C) ARA) and complex selection pressure ((D) HSM, (E) LSM, and (F) GXA) regimes. Scatter plots indicate the specific growth rate observed at each passage according to cumulative cell division (CCD). Line plots represent the moving average trendline for every 5 points. For GXA, only the trendline from the xylose flasks, which showed the only increasing trend, was depicted. The *x*- and *y*-axes indicate CCD and specific growth rate (h^{-1}), respectively. Each color represents replicated ALE experiments. Source data are provided as a Source Data file.

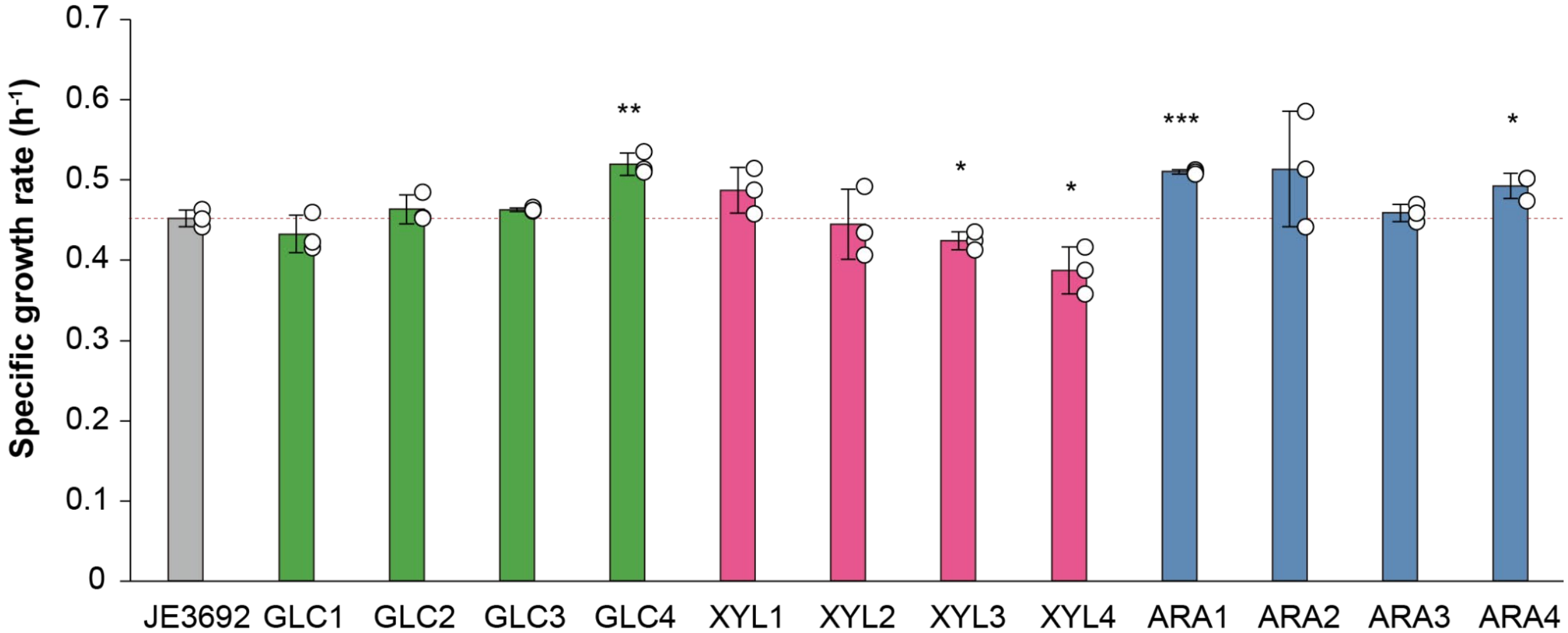

Supplementary Figure 2. Specific growth rates of strains evolved under simple selection pressure in mixed-sugar conditions with 4 g/L of each sugar

The *x*- and *y*-axes indicate strains and specific growth rate (h^{-1}), respectively. The gray, green, pink, and blue boxes represent JE3692, GLC, XYL, and ARA isolates, respectively. Red dotted lines indicate the growth rate of the JE3692 strain. Bars represent mean specific growth rates from three biological replicates (n = 3), and error bars indicate the standard deviations. White dots overlaid on the bar graphs represent individual biological replicates. Statistical significance was assessed using two-sided Student's T-tests assuming equal variances. No adjustment was made for multiple comparisons. *, **, and *** indicate $p < 0.05$, $p < 0.01$, and $p < 0.001$, respectively. Bars without asterisks represent values that are not statistically significantly different from those of the JE3692 strain. Source data are provided as a Source Data file.

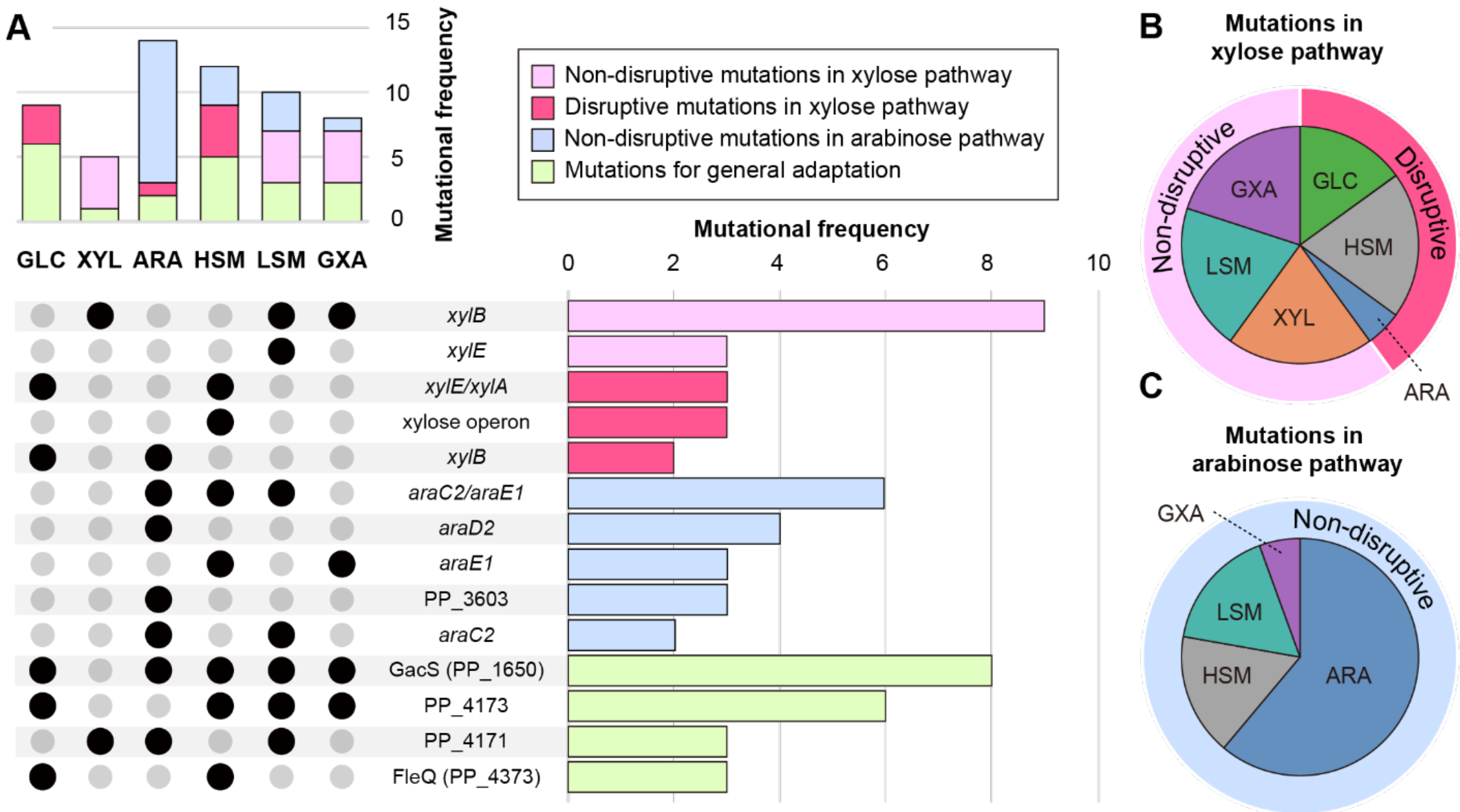

Supplementary Figure 3. Distribution of mutational frequencies across different ALE groups

(A) UpSet plots to summarize the mutational patterns across the ALE groups. The top vertical bar chart represents the mutational frequency by ALE group. The bottom right horizontal bar chart represents the mutational frequency of the sets sharing mutational genes or regions, which are specified by black dots in the bottom left. Pie plots showing the mutational frequencies identified in the (B) xylose and (C) arabinose pathways across each ALE group and their impact on functionality. The color code of the outer pie plots in panel B and C is the same as in the bar plots in panel A.

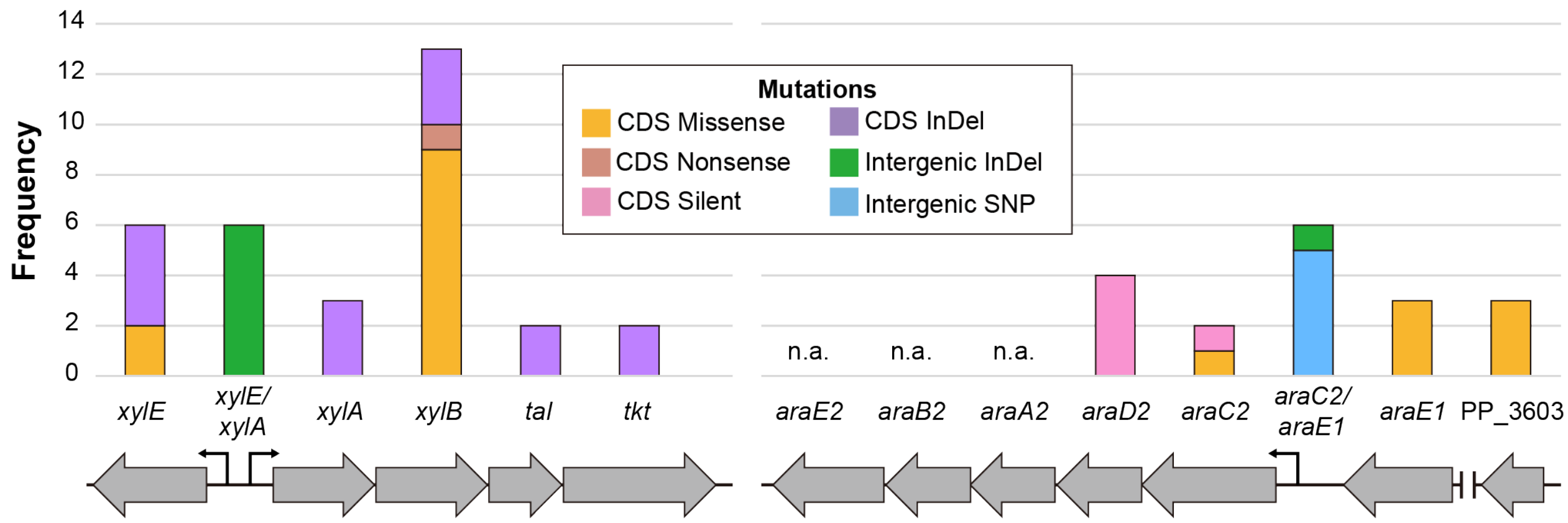

Supplementary Figure 4. Mutational frequency distribution across genetic regions of the xylose and arabinose catabolic pathways

Frequency of mutational types in regions related to catabolism of xylose and arabinose. Different colors represent different mutation types detailed in the figure key. The *x*- and *y*-axes indicate genetic region and mutational frequency. The arrows below the *x*-axis depict the genetic context.

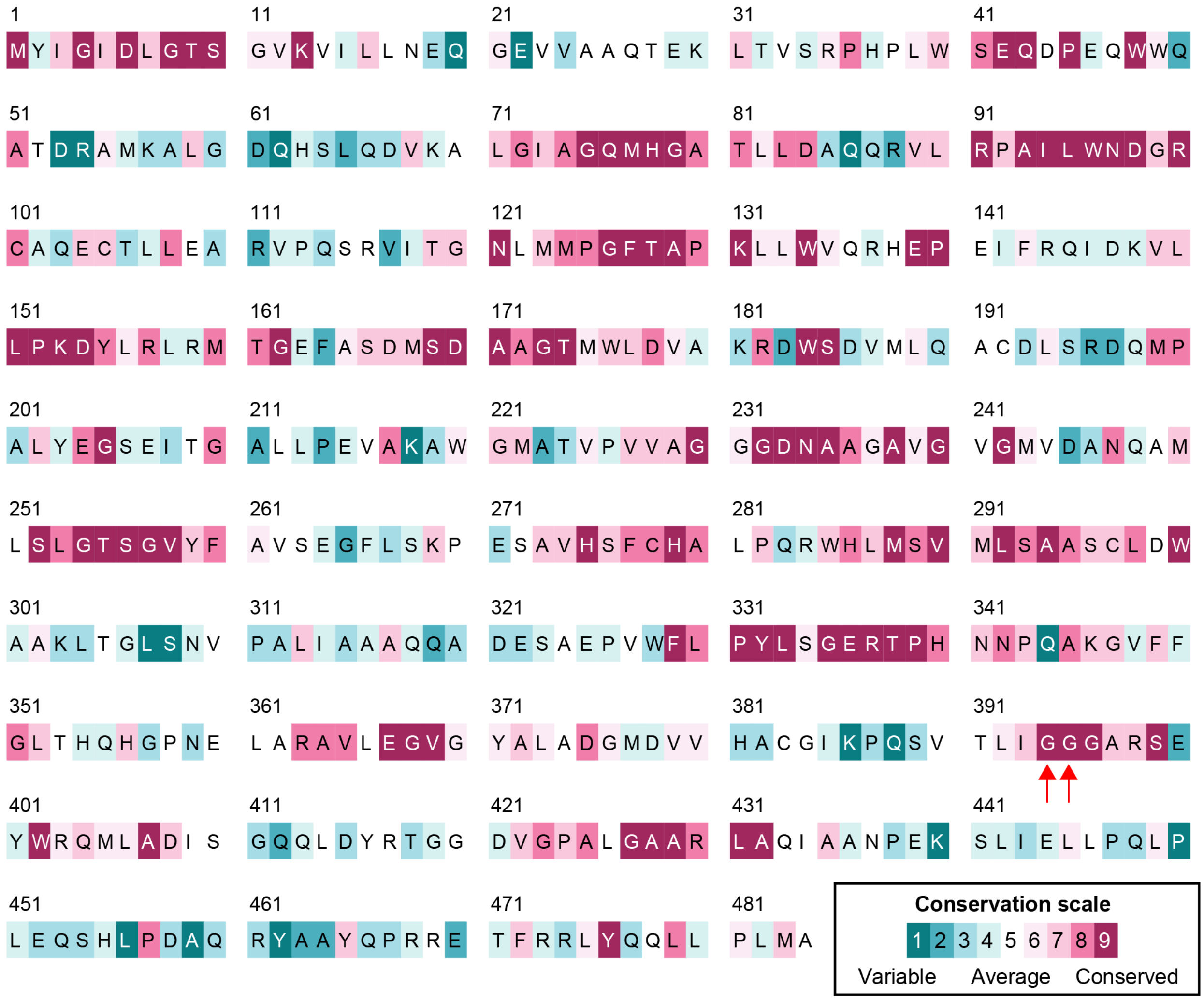

Supplementary Figure 5. Conservation analysis of XylB

The degree of amino acid conservation is shown by the coloring scheme. The color intensity increases based on conservation grades; e.g. turquoise indicates variable sites, white indicates average sites, maroon indicates evolutionarily conserved sites. The convergently mutated residues (395 and 396) are indicated by red arrows.

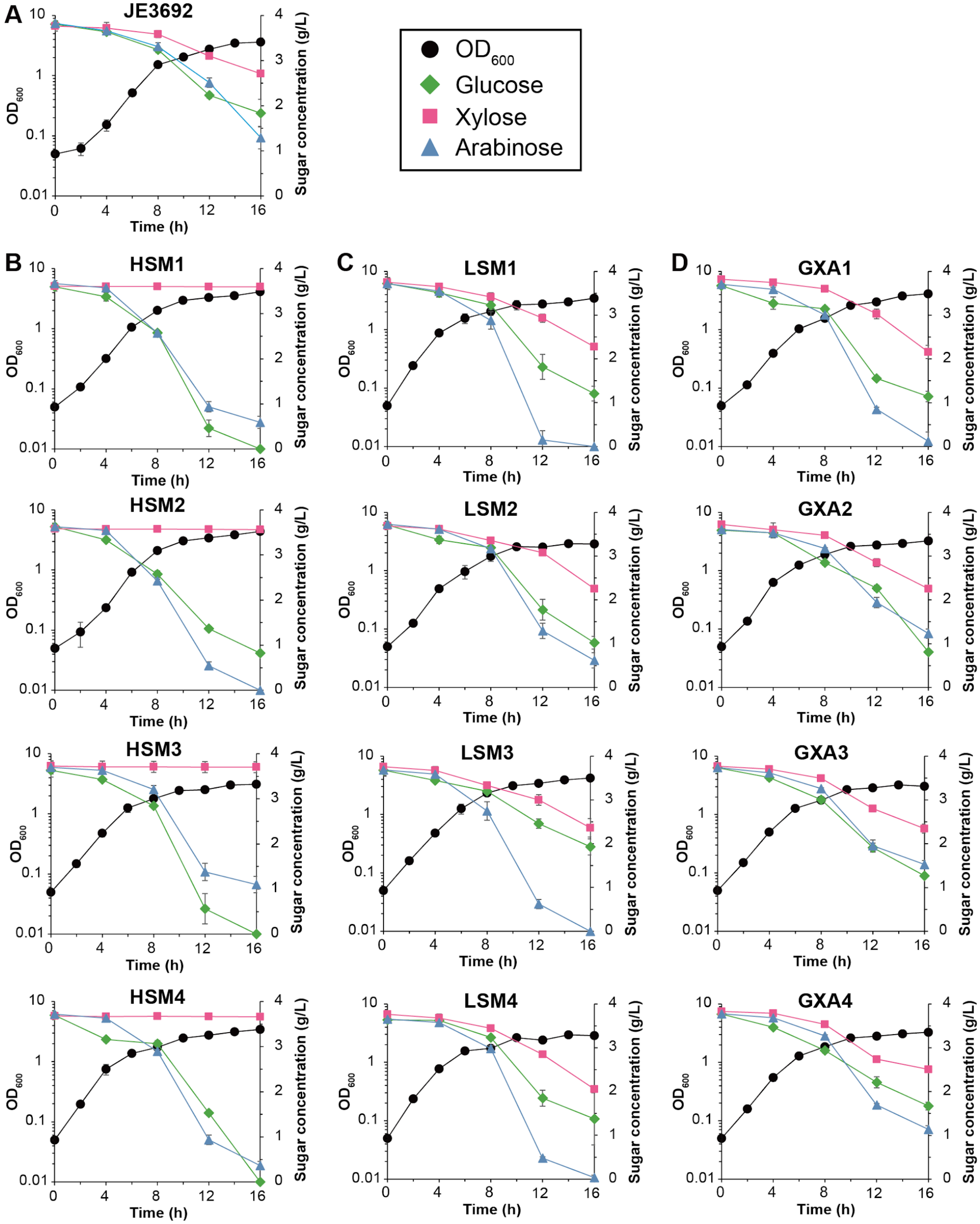

Supplementary Figure 6. Time-course profiles of cell growth and sugar consumption of the parental strain and strains evolved under complex selection pressures in mixed-sugar conditions

Time-course profiles of cell growth and sugar consumption for isolates from (A) parental strain JE3692 and the isolates evolved under (B) HSM, (C) LSM, and (D) GXA regimes in mixed-sugar conditions. Symbols: black circle, OD_{600}; green diamond, glucose; pink square, xylose; blue triangle, arabinose. Each point represents mean values from three biological replicates (n = 3), and error bars indicate the standard deviations. Source data are provided as a Source Data file.

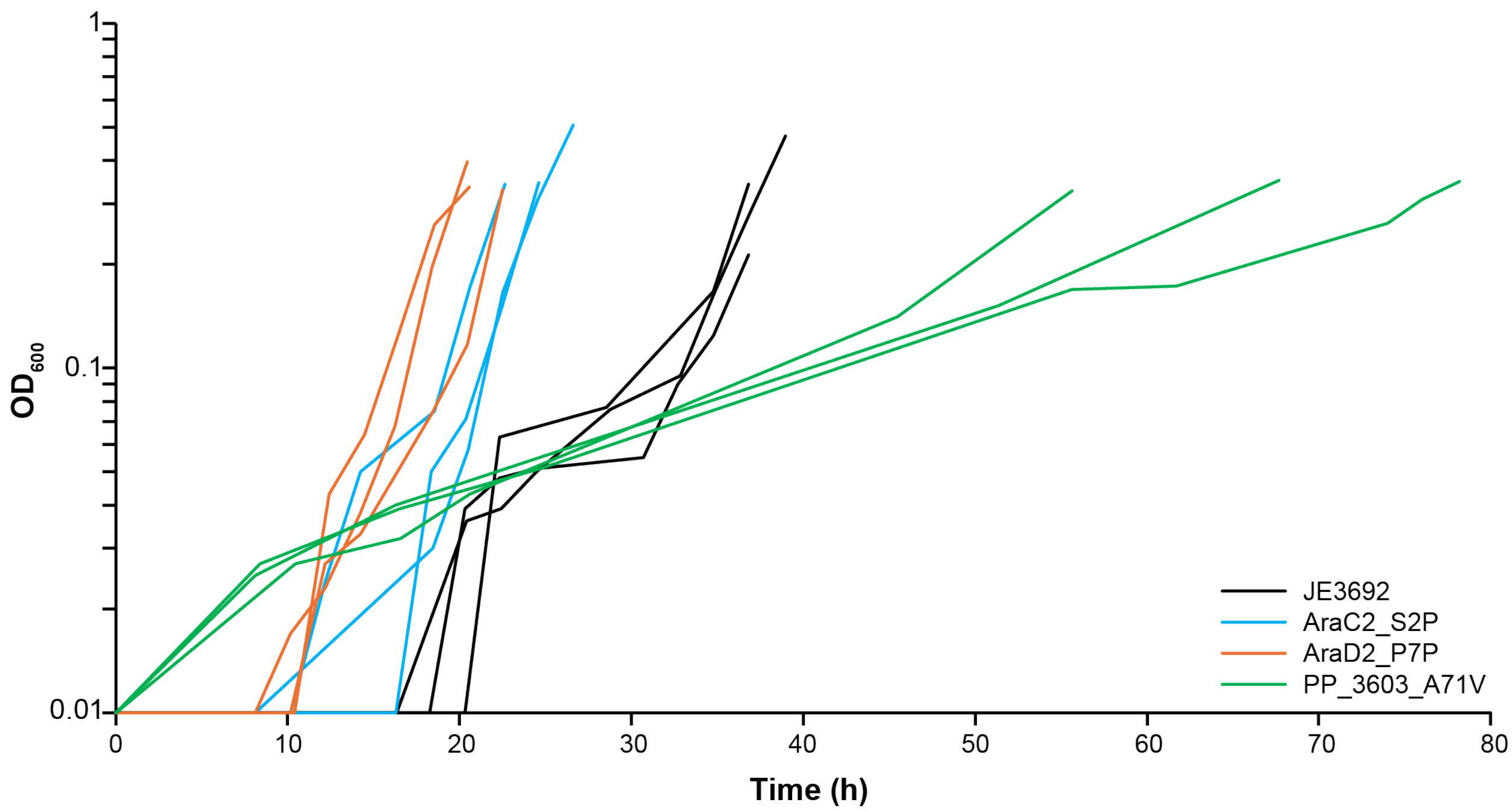

Supplementary Figure 7. Growth profiles under arabinose conditions for strains carrying key mutations

Growth profiles of the JE3692, AraC2_S2P, AraD2_P7P, and PP_3603_A71V strains in minimal medium supplemented with 4 g/L arabinose. The x- and *y*-axes indicate time (h) and cell biomass (OD_{600}), respectively. The cultures were conducted with three biological replicates (n = 3). Source data are provided as a Source Data file.

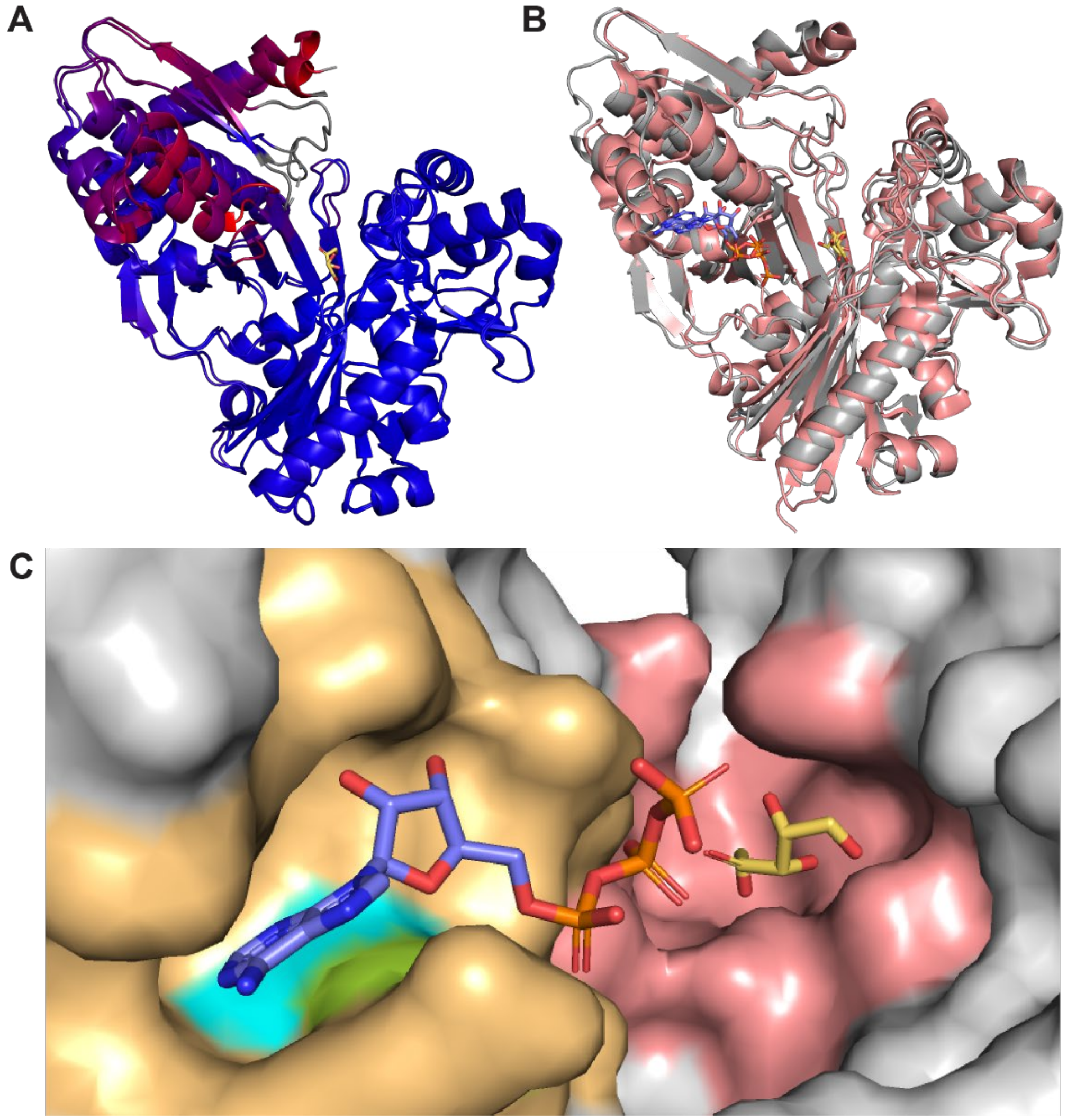

Supplementary Figure 8. Generation of an AlphaFold3 model of *E. coli* XylB with ATP and D-xylulose

(A) Alignment of the *E. coli* XylB crystal structure (PDB: 2ITM) complexed with D-xylulose (XUL) with the AlphaFold3 (AF3) model (all-atom RMSD = 0.625 Å), showing close alignment at the N-terminal region and the XUL binding site. This structural correspondence supports transferring the XUL ligand from the 2ITM crystal structure to the AF3 model. Structures are colored by RMSD, with blue indicating lower deviation and red indicating higher deviation. (B) Alignment of a XylB homolog crystal structure from *Lactobacillus acidophilus* (PDB: 3LL3) complexed with ATP and XUL with the AlphaFold3 structure of *E. coli* XylB with ATP docked (all-atom RMSD = 1.573 Å). The conserved placement of ATP in 3LL3 supports the ATP docking generated by the AF3 web server. (C) AF3 model of *E. coli* XylB with ATP (positioned by AF3 docking) and XUL (transferred from the 2ITM structure). Residues within 6 Å are highlighted for ATP (orange) and XUL (red). The mutated residues G395 and G396, shown in cyan and green, respectively, are located in the ATP-binding site. For visual clarity of all the displayed XylB structures, only one monomer of the dimeric biological assembly is shown.

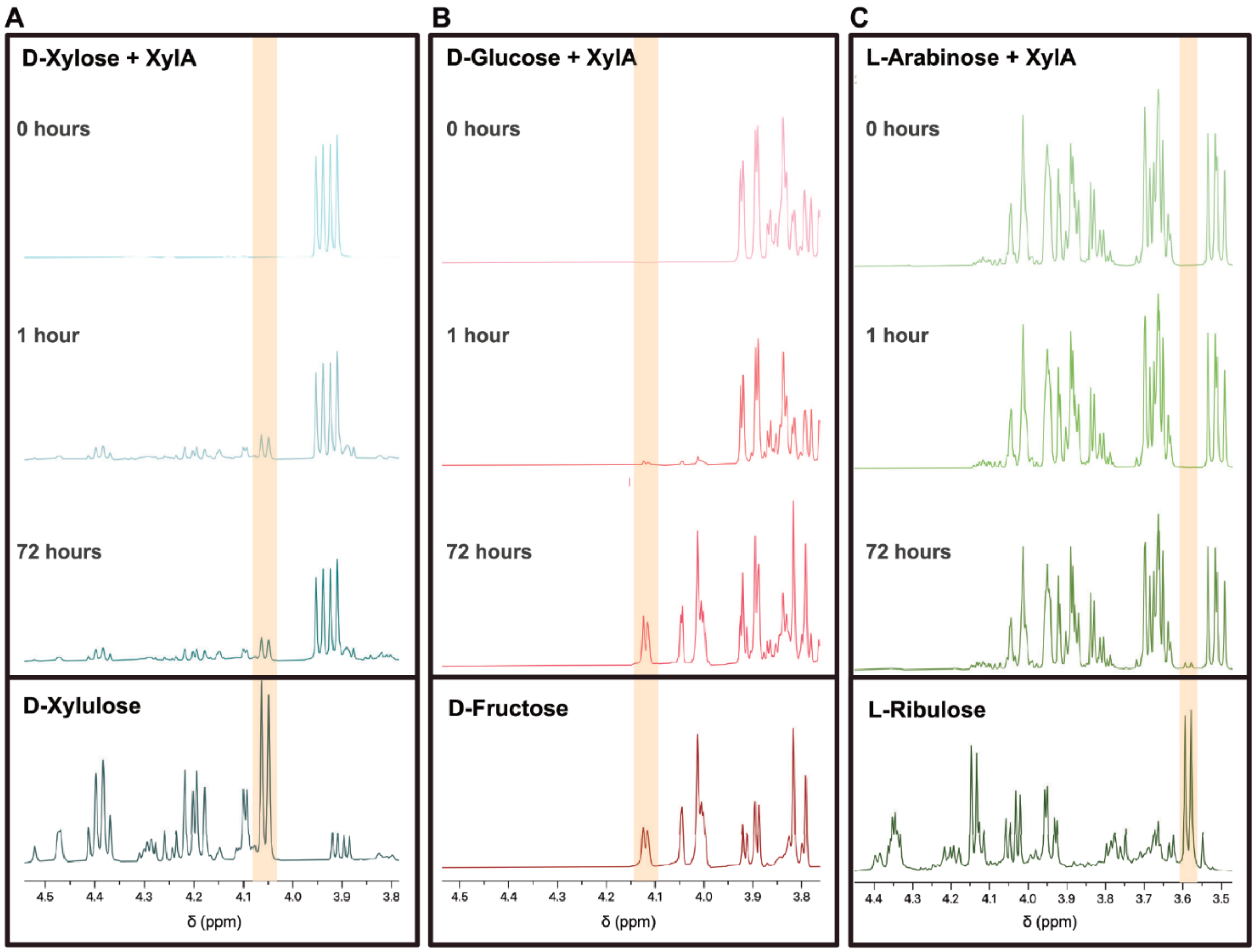

D

	Peak		Integration			Concentration (mM)		
	**Center (ppm)**	**No. Hs**	**0 hours**	**1 hour**	**72 hours**	**0 hours**	**1 hour**	**72 hours**
**D-Xylulose**	4.056	2	N.D.	0.22	0.22	N.D.	1.3	1.3
**D-Fructose**	4.120	2	N.D.	0.05	0.74	N.D.	0.17	2.2
**L-Ribulose**	3.590	≥1	N.D.	N.D.	0.13	N.D.	N.D.	≤0.7

Supplementary Figure 9. NMR analysis of XylA activity on various sugar substrates

Time-course proton NMR spectra of reactions containing recombinant XylA and either (A) D-xylose, (B) D-glucose, or (C) L-arabinose over 72 hours. Each panel includes spectra at 0, 1, and 72 hours after XylA addition. Spectra for D-xylulose, D-fructose, and L-ribulose standards are shown for comparison. The shaded regions highlight a unique product peak for quantification against an internal standard. (D) Summary of representative product peaks including chemical shift, number of hydrogens, signal integration, and calculated concentrations based on comparison to an internal standard at 1 hour and 72 hours. D-xylulose formation from D-xylose is observed rapidly and remains stable. D-fructose accumulates gradually in D-glucose reactions. Minor L-ribulose formation is detected from L-arabinose. "N.D." indicates signal not detected above noise. No conversion was observed for samples without XylA (data not shown).

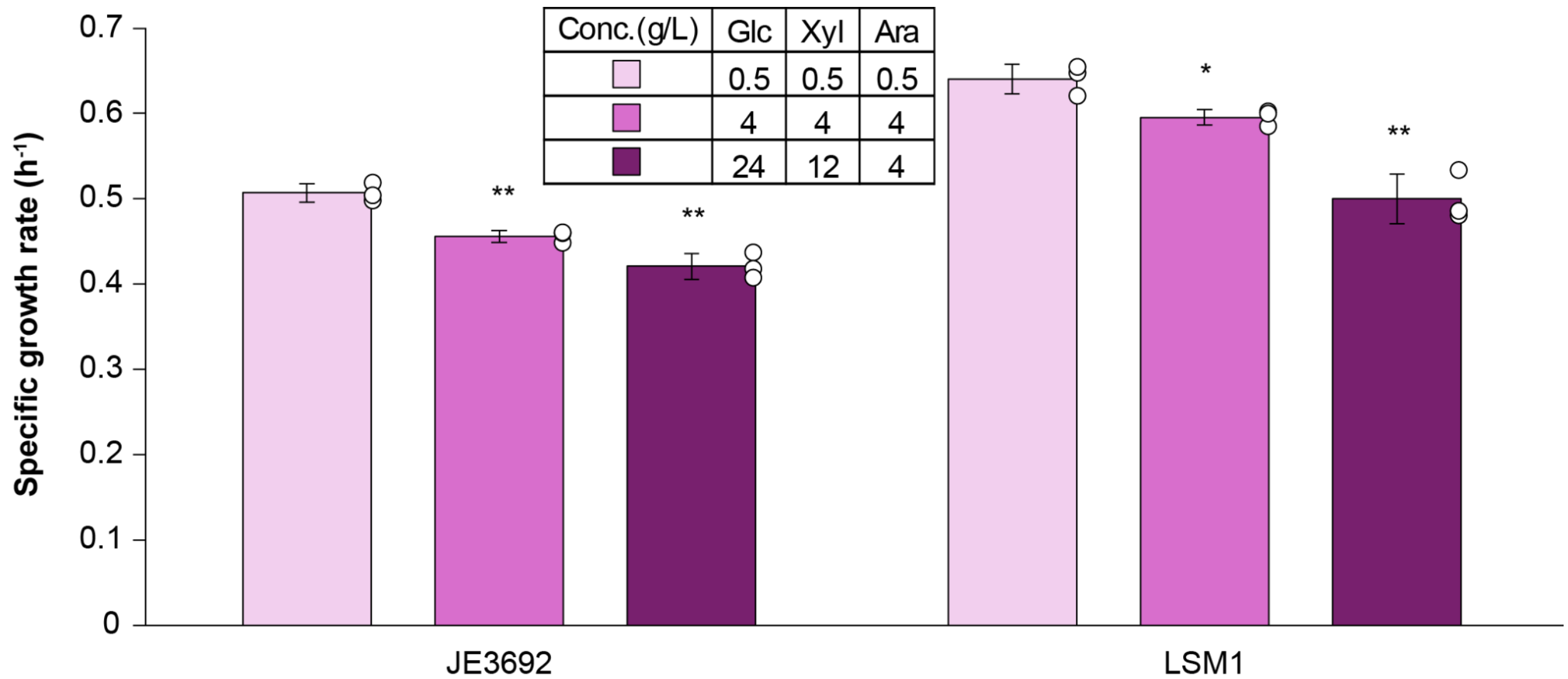

Supplementary Figure 10. Comparison of specific growth rates under the mixed-sugar conditions across different sugar concentrations

Specific growth rates of the JE3692 and LSM1 strains under mixed-sugar conditions: 0.5 g/L each, 4 g/L each, and mock hydrolysate (24 g/L glucose, 12 g/L xylose, and 4 g/L arabinose). Bars represent mean specific growth rates from three biological replicates ($n = 3$), and error bars indicate the standard deviations. White dots overlaid on the bar graphs represent individual biological replicates. Statistical significance was assessed using two-sided Student's T-tests assuming equal variances. No adjustment was made for multiple comparisons. * and ** indicate $p < 0.05$ and $p < 0.01$, respectively. Source data are provided as a Source Data file.

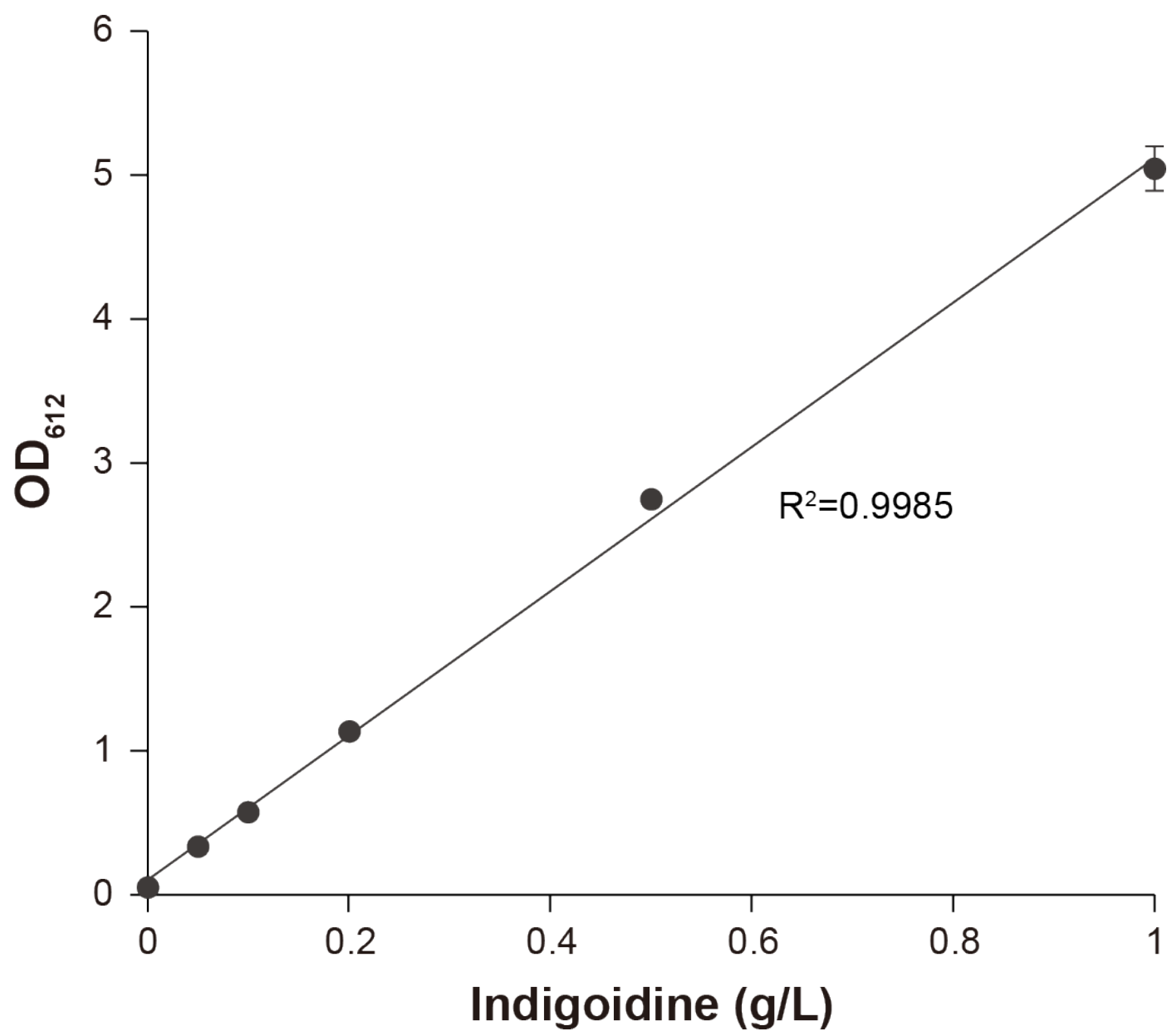

Supplementary Figure 11. Standard curve for indigoidine quantification

Indigoidine was dissolved at various concentrations in the minimal medium and the absorbance at 612 nm was measured. Error bars indicate standard deviations of three technical replicates. Source data are provided as a Source Data file.

Supplementary Table 1. Classification of ALE regimes and their selection characteristics

Classification	ALE regime	Condition	Selection characteristics
Simple selection pressure (Single substrate)	GLC	Glucose 4 g/L	Served as the baseline control group for condition-specific adaptation.
	XYL	Xylose 4 g/L	
	ARA	Arabinose 4 g/L	
Complex selection pressure (Mixed substrates)	HSM	Glucose 4 g/L+<br>Xylose 4 g/L+<br>Arabinose 4 g/L<br>(Selective consumption allowed)	Metabolism-Driven Temporal Shifts: Internal shifts in selection pressure driven by the cells' differential consumption rates within a passage.
	LSM	Glucose 0.5 g/L+<br>Xylose 0.5 g/L+<br>Arabinose 0.5 g/L<br>(Complete consumption mandated)	
Complex selection pressure (Alternating substrates)	GXA	Glucose 4 g/L/<br>Xylose 4 g/L/<br>Arabinose 4 g/L<br>(Alternating supply)	Experimenter-Driven Sequential Pressure: Intentional, alternating substrate replacement across successive passages through external intervention.

Supplementary Table 2. Summary of the ALE experiment results

ALE regime	Initial growth rate (h^{-1})	Replicate	# of passages	# of generations	Cumulative cell divisions (CCD, 10^{11})	Growth rate of the end-point population (h^{-1})
GLC	0.42 ± 0.04	1	97	634	52.2	0.62
		2	98	639	52.0	0.63
		3	97	631	53.2	0.56
		4	95	618	51.5	0.57
XYL	0.20 ± 0.01	1	65	394	36.6	0.44
		2	67	413	39.6	0.36
		3	70	417	33.1	0.44
		4	69	413	39.3	0.43
ARA	0.14 ± 0.02	1	74	479	42.3	0.40
		2	78	491	44.1	0.44
		3	81	519	43.7	0.48
		4	80	514	43.8	0.51
HSM	0.46 ± 0.04	1	105	687	58.9	0.58
		2	106	692	59.1	0.52
		3	107	692	59.2	0.66
		4	109	709	61.5	0.58
LSM	0.46 ± 0.04	1	103	671	38.8	0.58
		2	96	627	37.1	0.68
		3	106	689	42.8	0.62
		4	95	617	37.5	0.61
GXA	Glucose: 0.42 ± 0.04<br>Xylose: 0.20 ± 0.01<br>Arabinose: 0.14 ± 0.02	1	63	409	32.4	Glucose: 0.67<br>Xylose: 0.25<br>Arabinose: 0.15
		2	70	447	35.4	Glucose: 0.65<br>Xylose: 0.37<br>Arabinose: 0.21
		3	68	441	34.9	Glucose: 0.57<br>Xylose: 0.44<br>Arabinose: 0.22
		4	68	437	33.5	Glucose: 0.51<br>Xylose: 0.40<br>Arabinose: 0.27

Supplementary Table 3. List of strains and plasmids used in this study

Name	Description	Source
**Strains**		
JE212	*P. putida* KT2440 Δ*hsdR::Bxb1int-attB* Δ*gcd*	1
JE3226	*P. putida* KT2440 Δ*hsdR::Bxb1int-attB* Δ*gcd* Δ*ampC::PxylE*-xylE:Ptac-xylAB:talB:tktA*	1
JE3285	*P. putida* KT2440 Δ*hsdR::Bxb1int-attB* Δ*gcd::PxylE-araE1:Ptac-araC2D2A2B2E2* Δ*ampC::PxylE*-xylE:Ptac-xylAB:talB:tktA*	1
JE3692	*P. putida* KT2440 Δ*hsdR::Bxb1int-attB* Δ*gcd::PxylE-araE1:Ptac-araC2D2A2B2E2* Δ*ampC::PxylE*-xylE:Ptac-xylAB:talB:tktA* Δ*crc*	1
BL21(DE3)	*E. coli* expression host for XylA and XylB	NEB
GLC1-4	Isolates from evolved lineages under GLC ALE regime	This study
XYL1-4	Isolates from evolved lineages under XYL ALE regime	This study
ARA1-4	Isolates from evolved lineages under ARA ALE regime	This study
HSM1-4	Isolates from evolved lineages under HSM ALE regime	This study
LSM1-4	Isolates from evolved lineages under LSM ALE regime	This study
GXA1-4	Isolates from evolved lineages under GXA ALE regime	This study
XylB_G395W	JE3692 *xylB* p.Gly395Trp	This study
XylE_Y179C	JE3692 *xylE* p.Tyr179Cys	This study
AraC2_S2P	JE3692 *AraC2* p.Ser2Pro	This study
AraD2_P7P	JE3692 *AraD2* p.Pro7Pro	This study
PP_3603_A71V	JE3692 PP_3603 p.Ala71Val	This study
JE3692_IND	JE3692/pTE252_IND	This study
LSM1_IND	LSM1/pTE252_IND	This study
**Plasmids**		
pET15b	Expression vector for XylA and XylB	Novagen
pEUK238	pET15b_XylA	This study
pEUK239	pET15b_XylB	This study
pEUK239_1	pET15b_XylB_G395R	This study
pEUK239_2	pET15b_XylB_G395W	This study
pNEG0020	Recombination plasmid XylB_G395W, based on pK18mobsacB	This study
pNEG0024	Recombination plasmid XylE_Y179C, based on pK18mobsacB	This study
pNEG0025	Recombination plasmid AraC2_S2P, based on pK18mobsacB	This study
pNEG0021	Recombination plasmid AraD2_P7P, based on pK18mobsacB	This study
pNEG0023	Recombination plasmid PP_3603_A71V, based on pK18mobsacB	This study
pTE251	pBBR1_*oriV_nptII_araC*_P_{BAD}_*sfp_bpsA*	2
pTE252	pBBR1_*oriV_aacC1_araC*_P_{BAD}_*sfp_bpsA*, a gentamicin-resistant variant of pTE251	This study
pJE443	pK18mobsacB Δ*ampC::*P_{xylE}-*xylE:*P_{tac}-*xylAB:talB:tktA* cassette, source of *tac* promoter	1
pTE252_IND	pTE252_pBBR1_*oriV_aacC1*_P_{tac}_*sfp_bpsA*	This study

Supplementary Table 4. Oligonucleotides used in this study

Name	Sequence (5'-3')
XylA_WT_F	GCCGCGCGGCAGCCATATGCAGGCCTACTTCGAC
XylA_WT_R	GTTAGCAGCCGGATCCTCACTTATCGAACAGGTAGTG
XylB_WT_F	GCCGCGCGGCAGCCATATGTACATTGGTATTGATTTGGG
XylB_WT_R	GTTAGCAGCCGGATCCTTAGGCCATCAGCGGC
XylB_G395R:1_F	CCTGATCGGCCGCGGCGCCCGGT
XylB_G395R:1_R	GTCACCGACTGTGGCTTGATG
XylB_G395W:2_F	CCTGATCGGCTGGGGCGCCCGGT
XylB_G395W:2_R	GTCACCGACTGTGGCTTGATGCCGC
XylB_recomb_ch_F	TTGGGTCCTGGTTATGCTGC
XylB_recomb_ch_R	CTTCCGGCAAATCGACAAGG
XylE_recomb_ch_F	TGCTTTTTTTCTGGCGCGTAATACAACACC
XylE_recomb_ch_R	AAACAAGCTTTGGGTTGTAGTGACGGAAGG
AraC2_recomb_ch_F	TGCTTTTTTTGGTCTGGATTCTGTGTTCGG
AraC2_recomb_ch_R	AAACAAGCTTTGAGAAAGTGGTGGAGGTCG
AraD2_recomb_ch_F	TGCTTTTTTTGACGGTCAATGGGAAGAGC
AraD2_recomb_ch_R	TCTAGCTCTAAAACAAGCTTTCCAGAATCGGACGAATGCC
PP3603_recomb_ch_F	TGCTTTTTTTGTGCTACCAGAACTACCCG
PP3603_recomb_ch_R	CTAGCTCTAAAACAAGCTTTGCAGGTGATCATAGCCAGG
ptac_sfp_bpsA_ins_F	TCTTATGACAACTTGACGGCTACATCATTCACGCCTGGAAGACGCAGACATTCC
ptac_sfp_bpsA_vec_R	GTGAATGATGTAGCCGTCAAGTTGTCATAA
ptac_sfp_bpsA_ins_R	TTCATATGTATATCTCCTTCTTAAAAGATCTTTTGAATTCAATTGTTATCCGCTCACAATTCCACACAT
ptac_sfp_bpsA_vec_F	GAATTCAAAAGATCTTTTAAGAAGGAGATATACATATGAAGATTTACG

Supplementary Table 5. Mutations in the starting strain JE3692

Gene	Protein	Modification	Mutation
PP_4387/ *flgE* (PP_4388)	Hypothetical protein/ flagellar hook protein	-GGC	Intergenic (-24/+44)
rluA (PP_1731)/ *minE* (PP_1732)	Ribosomal large subunit pseudouridine synthase/ cell division topological specificity factor	-C	Intergenic (-107/+43)
xylE/ *xylA*	D-xylose:proton symporter/ D-xylose isomerase	-T	Intergenic (-103/-105)

Reference genome: *P. putida* KT2440[3]

Supplementary Table 6. Whole-genome sequencing sample list

Strains	Bioproject	Biosample	ALEdb ID
JE3692	PRJNA1395279	SAMN54354527	A0_F0_I1_R1
GLC1	PRJNA1395279	SAMN54354528	A1_F83_I3_R1
GLC2	PRJNA1395279	SAMN54354529	A2_F83_I3_R1
GLC3	PRJNA1395279	SAMN54354530	A3_F81_I2_R1
GLC4	PRJNA1395279	SAMN54354531	A4_F78_I3_R1
XYL1	PRJNA1395279	SAMN54354532	A5_F53_I1_R1
XYL2	PRJNA1395279	SAMN54354533	A6_F57_I3_R1
XYL3	PRJNA1395279	SAMN54354534	A7_F62_I2_R1
XYL4	PRJNA1395279	SAMN54354535	A8_F59_I2_R1
ARA1	PRJNA1395279	SAMN54354536	A9_F65_I3_R1
ARA2	PRJNA1395279	SAMN54354537	A10_F67_I3_R1
ARA3	PRJNA1395279	SAMN54354538	A11_F71_I1_R1
ARA4	PRJNA1395279	SAMN54354539	A12_F70_I3_R1
HSM1	PRJNA1395279	SAMN54354540	A17_F89_I1_R1
HSM2	PRJNA1395279	SAMN54354541	A18_F91_I2_R1
HSM3	PRJNA1395279	SAMN54354542	A19_F93_I1_R1
HSM4	PRJNA1395279	SAMN54354543	A20_F93_I3_R1
LSM1	PRJNA1395279	SAMN54354544	A13_F87_I2_R1
LSM2	PRJNA1395279	SAMN54354545	A14_F87_I1_R1
LSM3	PRJNA1395279	SAMN54354546	A15_F91_I1_R1
LSM4	PRJNA1395279	SAMN54354547	A16_F83_I2_R1
GXA1	PRJNA1395279	SAMN54354548	A25_F53_I2_R1
GXA2	PRJNA1395279	SAMN54354549	A26_F59_I3_R1
GXA3	PRJNA1395279	SAMN54354550	A27_F59_I1_R1
GXA4	PRJNA1395279	SAMN54354551	A28_F59_I2_R1

Supplementary References

1. Elmore, J. R. et al. Engineered *Pseudomonas putida* simultaneously catabolizes five major components of corn stover lignocellulose: Glucose, xylose, arabinose, p-coumaric acid, and acetic acid. *Metab. Eng.* **62**, 62–71 (2020).
2. Gauttam, Rahul, et al. Development of genetic tools for heterologous protein expression in a pentose-utilizing environmental isolate of *Pseudomonas putida*. *Microbial Biotechnology* **16**, 645-661 (2023).
3. Lim, Hyun Gyu, et al. Generation of ionic liquid tolerant *Pseudomonas putida* KT2440 strains via adaptive laboratory evolution. *Green Chemistry* **22**, 5677-5690 (2020).
