# Supplementary material for: Simultaneous optimization of lignocellulosic sugar catabolism via systematic laboratory evolution under complex selection pressure
Source: Nat Commun. 2026 Jul 29;17:9194. doi: 10.1038/s41467-026-75974-x (PMC13522501; doi:10.1038/s41467-026-75974-x)
Supplement: Supplementary file 2 [file 41467_2026_75974_MOESM2_ESM.pdf]

Description of Additional Supplementary Data Files

File Name: Supplementary Data 1
Description: Summarized mutational information

File Name: Supplementary Data 2
Description: Summarized proteomic information
