# Supplementary material for: Simultaneous optimization of lignocellulosic sugar catabolism via systematic laboratory evolution under complex selection pressure
Source: Nat Commun. 2026 Jul 29;17:9194. doi: 10.1038/s41467-026-75974-x (PMC13522501; doi:10.1038/s41467-026-75974-x)
Supplement: Supplementary file 3 [file 41467_2026_75974_MOESM5_ESM.pdf]

# nature portfolio

Corresponding author(s): Adam M. Feist

Last updated by author(s): Jun 25, 2026

# Reporting Summary

Nature Portfolio wishes to improve the reproducibility of the work that we publish. This form provides structure for consistency and transparency in reporting. For further information on Nature Portfolio policies, see our Editorial Policies and the Editorial Policy Checklist.

## Statistics

For all statistical analyses, confirm that the following items are present in the figure legend, table legend, main text, or Methods section.

| n/a | Confirmed | |
|---|---|---|
| ☐ | ☒ | The exact sample size (*n*) for each experimental group/condition, given as a discrete number and unit of measurement |
| ☐ | ☒ | A statement on whether measurements were taken from distinct samples or whether the same sample was measured repeatedly |
| ☐ | ☒ | The statistical test(s) used AND whether they are one- or two-sided<br>*Only common tests should be described solely by name; describe more complex techniques in the Methods section.* |
| ☒ | ☐ | A description of all covariates tested |
| ☒ | ☐ | A description of any assumptions or corrections, such as tests of normality and adjustment for multiple comparisons |
| ☐ | ☒ | A full description of the statistical parameters including central tendency (e.g. means) or other basic estimates (e.g. regression coefficient) AND variation (e.g. standard deviation) or associated estimates of uncertainty (e.g. confidence intervals) |
| ☒ | ☐ | For null hypothesis testing, the test statistic (e.g. *F*, *t*, *r*) with confidence intervals, effect sizes, degrees of freedom and *P* value noted<br>*Give P values as exact values whenever suitable.* |
| ☒ | ☐ | For Bayesian analysis, information on the choice of priors and Markov chain Monte Carlo settings |
| ☒ | ☐ | For hierarchical and complex designs, identification of the appropriate level for tests and full reporting of outcomes |
| ☒ | ☐ | Estimates of effect sizes (e.g. Cohen's *d*, Pearson's *r*), indicating how they were calculated |

*Our web collection on statistics for biologists contains articles on many of the points above.*

## Software and code

Policy information about availability of computer code

| | |
|---|---|
| Data collection | None used |
| Data analysis | DIA-NN (https://github.com/vdemichev/DiaNN)<br>AlphaFold3 (https://github.com/google-deepmind/alphafold3) |

For manuscripts utilizing custom algorithms or software that are central to the research but not yet described in published literature, software must be made available to editors and reviewers. We strongly encourage code deposition in a community repository (e.g. GitHub). See the Nature Portfolio guidelines for submitting code & software for further information.

## Data

Policy information about availability of data

All manuscripts must include a data availability statement. This statement should provide the following information, where applicable:

- Accession codes, unique identifiers, or web links for publicly available datasets
- A description of any restrictions on data availability
- For clinical datasets or third party data, please ensure that the statement adheres to our policy

The raw genome-sequencing files are available at the National Center for Biotechnology Information (NCBI) Sequence Read Archive (SRA) under accession code PRJNA1395279 (Supplementary Table 6) [https://www.ncbi.nlm.nih.gov/bioproject/PRJNA1395279/]. The variant-calling data is available in ALEdb v1.093 under project name Pputida_complex_ALE [https://aledb.org/ale/project/259/]. The generated mass spectrometry proteomics data have been deposited in the

ProteomeXchange Consortium via the PRIDE94 partner repository with the dataset identifier PXD055215 [https://proteomecentral.proteomexchange.org/ui?pxid=PXD055215]. Protein structures used in this work include PDB IDs 2ITM [https://www.rcsb.org/structure/2ITM] and 3LL3 [https://www.rcsb.org/structure/3LL3]. The P. putida KT2440 UniProt proteome FASTA used for DIA-NN searches was obtained from UniProt proteome UP000000556 [https://www.uniprot.org/proteomes/UP000000556].

## Research involving human participants, their data, or biological material

Policy information about studies with human participants or human data. See also policy information about sex, gender (identity/presentation), and sexual orientation and race, ethnicity and racism.

| | |
|---|---|
| Reporting on sex and gender | Not applicable |
| Reporting on race, ethnicity, or other socially relevant groupings | Not applicable |
| Population characteristics | Not applicable |
| Recruitment | Not applicable |
| Ethics oversight | Not applicable |

Note that full information on the approval of the study protocol must also be provided in the manuscript.

# Field-specific reporting

Please select the one below that is the best fit for your research. If you are not sure, read the appropriate sections before making your selection.

☒ Life sciences ☐ Behavioural & social sciences ☐ Ecological, evolutionary & environmental sciences

For a reference copy of the document with all sections, see nature.com/documents/nr-reporting-summary-flat.pdf

# Life sciences study design

All studies must disclose on these points even when the disclosure is negative.

| | |
|---|---|
| Sample size | Adaptive laboratory evolution was performed with four independent replicates. Cultivation data were collected from two or three biological replicates, and proteomic analyses were conducted using four biological replicates. All other experiments were performed with three biological replicates. |
| Data exclusions | No data was excluded. |
| Replication | All attempts at replication were successful. |
| Randomization | Randomization was not applicable, as experimental groups were defined by strain or condition. |
| Blinding | Blinding was not performed because the experiments involved objective quantitative measurements and group identity could not be concealed. |

# Reporting for specific materials, systems and methods

We require information from authors about some types of materials, experimental systems and methods used in many studies. Here, indicate whether each material, system or method listed is relevant to your study. If you are not sure if a list item applies to your research, read the appropriate section before selecting a response.

### Materials & experimental systems

| n/a | Involved in the study | |
|---|---|---|
| ☒ | ☐ | Antibodies |
| ☒ | ☐ | Eukaryotic cell lines |
| ☒ | ☐ | Palaeontology and archaeology |
| ☒ | ☐ | Animals and other organisms |
| ☒ | ☐ | Clinical data |
| ☒ | ☐ | Dual use research of concern |
| ☒ | ☐ | Plants |

### Methods

| n/a | Involved in the study | |
|---|---|---|
| ☒ | ☐ | ChIP-seq |
| ☒ | ☐ | Flow cytometry |
| ☒ | ☐ | MRI-based neuroimaging |

## Plants

| | |
|---|---|
| Seed stocks | Not applicable |
| Novel plant genotypes | Not applicable |
| Authentication | Not applicable |
