# Supplementary material for: Simultaneous optimization of lignocellulosic sugar catabolism via systematic laboratory evolution under complex selection pressure
Source: Nat Commun. 2026 Jul 29;17:9194. doi: 10.1038/s41467-026-75974-x (PMC13522501; doi:10.1038/s41467-026-75974-x)
Supplement: Supplementary file 4 [file 41467_2026_75974_MOESM6_ESM.pdf]

nature portfolio

Peer Review File

Simultaneous optimization of lignocellulosic sugar catabolism via systematic laboratory evolution under complex selection pressure

Corresponding Author: Dr Adam Feist

This file contains all reviewer reports in order by version, followed by all author rebuttals in order by version.

Version 0:

Reviewer comments:

Reviewer #1

(Remarks to the Author)
I co-reviewed this manuscript with one of the reviewers who provided the listed reports. This is part of the Nature Communications initiative to facilitate training in peer review and to provide appropriate recognition for Early Career Researchers who co-review manuscripts.

In this manuscript, Woo et al. investigate how different adaptive laboratory evolution (ALE) regimes shape the evolutionary stability and performance of engineered sugar catabolic pathways in Pseudomonas putida. Using an automated ALE platform, the authors systematically compare static single-sugar selection with several mixed- and sequential-sugar regimes and analyze the resulting evolutionary outcomes using phenotypic assays, whole-genome sequencing, proteomics, and targeted reverse engineering. The central finding is that carbon-limited mixed-sugar evolution promotes the emergence of "catabolic generalists" that stably retain and refine multiple heterologous pathways, whereas other regimes favor specialization or pathway loss.

The study addresses an important and timely problem in lignocellulosic biotechnology, namely the instability of heterologous catabolic pathways and the challenge of achieving robust co-utilization of mixed sugars. The experimental scale, the use of automation, and the comparative nature of the ALE regimes are clear strengths of the work. The results are largely consistent with evolutionary theory and prior ALE studies, but the systematic comparison across regimes in P. putida provides valuable insights. In principle, the study has the potential to be suitable for publication in Nature Communications.

However, in its current form, the manuscript does not fully exploit the potential of the experimental platform or the biological system. Several conclusions are insufficiently supported by data, key methodological details are missing or unclear, and some interpretations appear overstated. In particular, the evidence for true simultaneous sugar co-utilization, the definition and use of "dynamic" selection regimes, and the broader relevance of the evolved phenotypes for industrial biotechnology require further clarification and strengthening. Addressing the points below would substantially improve the rigor, clarity, and impact of the study.

Major comments

1) Definition and interpretation of "dynamic" ALE regimes: The manuscript repeatedly contrasts "static" and "dynamic" ALE conditions, and many conclusions hinge on this distinction. However, with the exception of the GXA regime (sequential glucose–xylose–arabinose exposure), the environments used in HSM and LSM are compositionally fixed within each passage. While substrate availability changes over time due to differential consumption, these conditions would more conventionally be described as multi-substrate static cultures rather than truly dynamic environments. Although this nuance is briefly acknowledged later in the manuscript, the terminology remains potentially misleading, especially given the central role of "dynamic selection" in the authors' narrative. I recommend clarifying this distinction early in the Introduction and being more precise throughout the text. One possibility would be to reserve "dynamic" primarily for the GXA regime and explicitly refer to HSM and LSM as mixed-substrate static conditions with different transfer criteria.

2) Evidence for sugar co-utilization versus sequential consumption: A key claim of the manuscript is the emergence of

catabolic generalists capable of improved utilization of mixed sugars. However, none of the main figures show time-resolved sugar consumption profiles for the evolved strains. Without such data, it is difficult to determine whether the improved phenotypes reflect true simultaneous co-utilization or simply faster sequential consumption (diauxic or triauxic growth). Given the importance of this distinction for both mechanistic interpretation and industrial relevance, I strongly recommend including time-course measurements of glucose, xylose, and arabinose consumption for the parental strain and representative evolved strains (especially LSM isolates) under mixed-sugar conditions.

3) Interpretation of LSM as a general strategy for industrial applications: The carbon-limited mixed-sugar (LSM) regime appears to be the most successful at maintaining multiple pathways, yet it operates at very low total sugar concentrations (1.5 g/L). The manuscript does not sufficiently address how phenotypes evolved under such conditions would translate to industrially relevant processes, which typically involve much higher sugar loads, inhibitory compounds, and often fed-batch or continuous operation. The Discussion should more critically assess whether the generalist phenotype observed in LSM is expected to remain stable under high sugar concentrations or strong production-associated stress. Related to this, it would be useful to comment on whether high osmotic pressure in HSM (12 g/L total sugar) may itself act as a selective force. Without such discussion, the broader applicability of the findings remains uncertain.

4) Why did GXA not yield generalists? The sequential GXA regime is conceptually the most “dynamic” condition tested, yet it did not produce generalist strains comparable to those from LSM. This is an interesting and potentially insightful negative result, but it is not sufficiently explored. One plausible explanation is that sequential exposure allows compartmentalized, sugar-specific adaptations and retention of hierarchical regulation, rather than selection for concurrent pathway optimization. The authors should expand the Discussion to explore this point. Additional evidence, such as growth or consumption profiles of GXA isolates in mixed-sugar media, would further strengthen this interpretation.

5) Clonal isolation strategy and population-level effects: The manuscript states that clonal isolates were obtained from end-point populations, but the isolation strategy is not clearly described. It is unclear how many clones per lineage were analyzed, how they were selected, and whether they are representative of the evolved populations. This is particularly important in mixed-sugar environments, where population-level solutions or polymorphisms may arise. The authors should clarify the clonal selection procedure and discuss the possibility that some population-level benefits or trade-offs may not be captured by analyzing a single isolate per lineage.

6) Choice and limitations of the xylose isomerase pathway: The instability and fitness cost of the E. coli xylose isomerase pathway play a central role in the study’s conclusions. While the choice of this pathway is understandable given prior work, it would strengthen the manuscript to more explicitly acknowledge alternative strategies, such as the Weimberg pathway, and discuss why the isomerase route was chosen despite its known drawbacks.

7) Methodological clarity and reproducibility of ALE experiments: Key details of the ALE experiments are insufficiently described. These include culture volumes, vessel types, OD or time criteria for transfers, how sugar depletion was assessed in LSM, and how uptake rates were calculated. Given that the ALE platform is central to the study, I recommend expanding the Methods to include a dedicated subsection describing the ALE setup in a level of detail appropriate for reproducibility.
The manuscript raises several other interesting questions that the authors might consider addressing in the Discussion section:

Could better indigoidine production be obtained solely through the co-utilization of XYL and GLC? It appears the major contribution actually stems from ARA catabolism.

What would be the optimal industrial bioprocess scenario, considering that high sugar loads are clearly unsuitable for maintaining generalist phenotypes? Continuous systems may not be viable either, as strains tend to favor the carbon source that allows for the fastest growth and minimal metabolic burden.

Could a synthetic consortium of P. putida specialist strains outperform the current setup?

How long would the best generalist clone, LSM1, retain its beneficial properties and sugar co-utilization capacity if exposed to different selection pressures (e.g., high sugar concentrations or the stress of indigoidine production)? Would some of the top clones maintain these properties longer than others?

Why was ALE focused strictly on growth rather than being linked to the production of the target metabolite(s)?

MINOR COMMENTS

1) Line 105-107: Please, add concentrations of sugars used individually.
2) Figure 2D: Mutations, Monsense should be nonsense
3) Line 108: Any explanation for the considerable reduction in growth rate?
4) Line 120-121: Please, clarify the precise criteria for OD and sugar depletion-based transfers.
5) Line 131-133: Clarify how were these clonal isolates selected.
6) Line 181: “motivating alternative ALE approaches” this was already part of the original hypothesis? If so, the motivation should have been the hypothesis itself.
7) Line 246: As mentioned previously since mixed-sugar conditions might select for a better performing population and that fact might be lost when analysing a singe HSM isolate. Even if it dominates the population, the overall fitness of the whole population might be balanced by less abundant genotypes similar to real-world microbial consortia. This topic and potential

limitation sould be explored in the discussion.
8) Line 268: I did not understand why it is “resequencing” and not just sequencing.
9) Line 291 and 293: “HSM mixed-sugar conditions” HSM is highlighted in the caption but all conditions HSM, LSM and GXA are presented in the figure 3 D and E.
10) Line 307: It would be interesting to see the effect of this mutation in Xylose and not just under Arabinose. Considering this mutation then does not appear under LSM, it seems it could be detrimental to xylose usage. If the authors have this data perhaps would be nice to include in a supplementary figure.
11) Line 393-394: I appreciate the inclusion of growth profiles in C. More growth profiles should be presented earlier in the paper.
12) Line 461-463: The first part of sentence I agree with, but then is this evolutionary set-up good for industrial and robust applications? The limitations should also be discussed.
13) Lines 541-545: Missing details on how the isolates were selected in the end of ALE.

Reviewer #2

(Remarks to the Author)
I co-reviewed this manuscript with one of the reviewers who provided the listed reports. This is part of the Nature Communications initiative to facilitate training in peer review and to provide appropriate recognition for Early Career Researchers who co-review manuscripts.

Reviewer #3

(Remarks to the Author)
Review of the manuscript entitled
“Simultaneous optimization of lignocellulosic sugar catabolism via systematic laboratory evolution” by Woo et al. (Control number NCOMMS-26-005036).

Adaptive laboratory evolution (ALE) has emerged as a powerful tool in metabolic engineering, both for uncovering new design principles and for improving microbial production strains. Numerous superior phenotypes have been obtained through ALE, including enhanced growth rates and product titers, expanded substrate utilization toward non-native carbon sources, and improved tolerance to environmental stresses.
Lignocellulosic biomass represents the most abundant renewable feedstock for the sustainable production of fuels and chemicals. It consists of three major polymers: cellulose (a homopolymer of glucose), hemicellulose, and lignin. Hemicellulose is structurally heterogeneous and composed of various five- and six-carbon monosaccharides, including xylose, arabinose, mannose, glucose, and galactose. Efficient and simultaneous utilization of these sugars remains a key challenge in strain engineering.
One of the central challenges in ALE is optimizing engineered microorganisms for complex feedstocks such as lignocellulosic biomass. In this context, Woo and coauthors demonstrate the application of distinct ALE regimes to evolve *Pseudomonas putida* previously engineered for xylose and arabinose catabolism. An automated ALE platform was employed to evolve the engineered strain under (i) static conditions with single selection pressures and (ii) dynamic regimes imposing selection on multiple sugars (xylose, arabinose, and glucose).
The authors show that different ALE regimes lead to distinct evolutionary outcomes, resulting in either catabolic specialists or generalists. Notably, the evolution of generalists occurred exclusively under carbon-limited mixed-sugar conditions requiring complete consumption of all three sugars. This finding provides valuable insight into how selective regimes shape metabolic breadth. Importantly, the study demonstrates that an evolved generalist isolate enhanced indigoidine production relative to the parental strain, highlighting the practical relevance of the approach. The strategy also shows strong potential for extension to more heterogeneous feedstocks, such as lignocellulosic hydrolysates.
Another important outcome of the study is the observation that evolution favoured mutations that reduced metabolic burden rather than maximizing enzymatic catalytic activity. This was exemplified in strains statically evolved for improved growth on xylose as a sole carbon source. Mutations in XylB (G395G and G395W) reduced ATP-binding affinity and consequently ATPase activity. Interestingly, the wild-type ATPase activity was associated with reduced growth compared to the mutants exhibiting lower activity. These results support the authors’ conclusion that, under selective pressure, evolution prioritizes minimizing energy and resource expenditure over maintaining maximal catalytic rates.

Overall, the manuscript presents solid, carefully executed work and makes an important contribution to the development of ALE strategies aimed at improving total sugar utilization in engineered microorganisms under mixed-sugar conditions.

Minor comments
1. Line 62: When strain JE3692 is first introduced, it would be helpful for a broader readership to clarify that it is a derivative of *P. putida* KT2440, a strain widely used in metabolic engineering.
2. Materials and Methods: The description of culture dilution during passaging is not sufficiently clear. Please specify the dilution factor used at each passage and indicate the approximate number of cells transferred (i.e., the effective bottleneck size).
3. Lines 414–426: Please refer explicitly to Supplementary Table S2 for the construction details of the plasmid expressing the indigoidine biosynthetic pathway. In line 422, please specify that plasmid pTE252_IND was transferred into JE3692 and LSM1.
4. Figure 4D: The legend refers to a red circle; however, only a red arrow is visible in the figure. Please clarify or correct.
5. Figure 5B: The concentrations of sugars used in the mock hydrolysate conditions (24 g/L glucose, 12 g/L xylose, 4 g/L

arabinose) are relatively high. Could the authors clarify whether these concentrations negatively affected *P. putida* growth?

Version 1:

Reviewer comments:

Reviewer #1

(Remarks to the Author)
The authors have addressed most of my previous comments. The revised manuscript is substantially improved: several claims have been toned down, important missing methodological details and sugar-consumption data have been added, and the limitations of the study are now discussed more openly. Overall, I consider the work technically strong, carefully executed, and sufficiently innovative.

That said, I still find some aspects of the manuscript somewhat overstated. The newly added data show that the improvement of the best “complex-condition” ALE isolate, LSM1, is only modest for glucose and xylose, which are the dominant sugars in most lignocellulosic hydrolysates. The strongest improvement appears to be in arabinose utilization, although arabinose usually represents a smaller fraction of such feedstocks.

I also remain unconvinced that the clone selection strategy fully captured the potential of the evolved populations. As only three fast-growing clones per condition were screened, it is possible that better-performing or more representative generalist clones were missed. Some outcomes of the ALE regimes also appear relatively predictable, particularly the tendency of high-sugar mixed conditions to favor specialization toward more readily used substrates…

One very interesting point in the study, highlighted also by the reviewer 3#, is that evolution often selected mutations that reduced the burden of the engineered pathways, rather than mutations that made the pathways work better. In other words, the cells seem to adapt by saving energy and resources, not necessarily by becoming better at using all sugars.

This raises an important question. If ALE often works in this way, how useful are standard or here presented ALE strategies for improving complex traits such as lignocellulosic sugar co-utilization? What alternative ALE setups could help select mutations that truly improve the combined use of glucose, xylose, and arabinose (and other lignocellulosic components), instead of simply reducing cellular burden or favouring one sugar over the others? I think these questions are important and should be discussed in the Discussion/Conclusion section of the manuscript.

These points do not, in my view, prevent publication, but they should be kept in mind when framing the generality and industrial relevance of the conclusions. I therefore support publication after minor textual adjustments.

I co-reviewed this manuscript with one of the reviewers who provided the listed reports. This is part of the Nature Communications initiative to facilitate training in peer review and to provide appropriate recognition for Early Career Researchers who co-review manuscripts.

Reviewer #2

(Remarks to the Author)
I co-reviewed this manuscript with one of the reviewers who provided the listed reports. This is part of the Nature Communications initiative to facilitate training in peer review and to provide appropriate recognition for Early Career Researchers who co-review manuscripts.

Reviewer #3

(Remarks to the Author)
The authors have satisfactorily addressed my previous concerns. The revised manuscript is clearly presented, and I have no additional comments.

Version 2:

Reviewer comments:

Reviewer #1

(Remarks to the Author)
Authors have adressed my comments.

Reviewer #2

(Remarks to the Author)
I co-reviewed this manuscript with one of the reviewers who provided the listed reports. This is part of the Nature

Communications initiative to facilitate training in peer review and to provide appropriate recognition for Early Career Researchers who co-review manuscripts.

Open Access This Peer Review File is licensed under a Creative Commons Attribution 4.0 International License, which permits use, sharing, adaptation, distribution and reproduction in any medium or format, as long as you give appropriate credit to the original author(s) and the source, provide a link to the Creative Commons license, and indicate if changes were made.
In cases where reviewers are anonymous, credit should be given to 'Anonymous Referee' and the source.
The images or other third party material in this Peer Review File are included in the article's Creative Commons license, unless indicated otherwise in a credit line to the material. If material is not included in the article's Creative Commons license and your intended use is not permitted by statutory regulation or exceeds the permitted use, you will need to obtain permission directly from the copyright holder.
To view a copy of this license, visit https://creativecommons.org/licenses/by/4.0/

Responses to reviewers' comments

We thank the reviewers for their careful evaluation and constructive comments, which helped improve the manuscript. Our response to each comment is presented below in blue. Changes made to the manuscript are highlighted in red.

Reviewer #1 (Remarks to the Author):

I co-reviewed this manuscript with one of the reviewers who provided the listed reports. This is part of the Nature Communications initiative to facilitate training in peer review and to provide appropriate recognition for Early Career Researchers who co-review manuscripts.

In this manuscript, Woo et al. investigate how different adaptive laboratory evolution (ALE) regimes shape the evolutionary stability and performance of engineered sugar catabolic pathways in *Pseudomonas putida*. Using an automated ALE platform, the authors systematically compare static single-sugar selection with several mixed- and sequential-sugar regimes and analyze the resulting evolutionary outcomes using phenotypic assays, whole-genome sequencing, proteomics, and targeted reverse engineering. The central finding is that carbon-limited mixed-sugar evolution promotes the emergence of “catabolic generalists” that stably retain and refine multiple heterologous pathways, whereas other regimes favor specialization or pathway loss.

The study addresses an important and timely problem in lignocellulosic biotechnology, namely the instability of heterologous catabolic pathways and the challenge of achieving robust co-utilization of mixed sugars. The experimental scale, the use of automation, and the comparative nature of the ALE regimes are clear strengths of the work. The results are largely consistent with evolutionary theory and prior ALE studies, but the systematic comparison across regimes in *P. putida* provides valuable insights. In principle, the study has the potential to be suitable for publication in Nature Communications.

However, in its current form, the manuscript does not fully exploit the potential of the experimental platform or the biological system. Several conclusions are insufficiently supported by data, key methodological details are missing or unclear, and some interpretations appear overstated. In particular, the evidence for true simultaneous sugar co-utilization, the definition and use of “dynamic” selection regimes, and the broader relevance of the evolved phenotypes for industrial biotechnology require further clarification and strengthening. Addressing the points below would substantially improve the rigor, clarity, and impact of the study.

We thank the reviewer for this positive feedback.

1) Definition and interpretation of “dynamic” ALE regimes: The manuscript repeatedly contrasts “static” and “dynamic” ALE conditions, and many conclusions hinge on this distinction. However, with the exception of the GXA regime (sequential glucose–xylose–arabinose exposure), the environments used in HSM and LSM are compositionally fixed within each passage. While substrate availability changes over time due to differential consumption, these conditions would

more conventionally be described as multi-substrate static cultures rather than truly dynamic environments. Although this nuance is briefly acknowledged later in the manuscript, the terminology remains potentially misleading, especially given the central role of “dynamic selection” in the authors’ narrative. I recommend clarifying this distinction early in the Introduction and being more precise throughout the text. One possibility would be to reserve “dynamic” primarily for the GXA regime and explicitly refer to HSM and LSM as mixed-substrate static conditions with different transfer criteria.

We agree that the terms "static" and "dynamic" could be misinterpreted, as the substrate composition in HSM and LSM is fixed at the start of each passage. To address this concern and more accurately reflect the logic of our experimental design, we have revised our classification to “simple vs. complex” selection pressure throughout the manuscript.

Under this new classification, simple selection pressure refers to environments where a single substrate is provided, specifically the GLC, XYL, and ARA regimes. In contrast, complex selection pressure encompasses environments involving multiple sugars, which we further distinguish based on the nature of substrate availability. The HSM and LSM regimes are now classified as mixed-substrate conditions, where temporal shifts in selection pressure are internally driven by the cells' differential consumption rates within a single passage. Meanwhile, the GXA regime is defined as an alternating-substrate condition, where the selection pressure is externally driven by the intentional replacement of substrates across successive passages.

In line with this refined terminology, we have changed the manuscript title to “Simultaneous optimization of lignocellulosic sugar catabolism via systematic laboratory evolution under complex selection pressure”. We also explicitly define this distinction in the Supplementary Table 1 and in the Results section (page 4, lines 128-133).

2) Evidence for sugar co-utilization versus sequential consumption: A key claim of the manuscript is the emergence of catabolic generalists capable of improved utilization of mixed sugars. However, none of the main figures show time-resolved sugar consumption profiles for the evolved strains. Without such data, it is difficult to determine whether the improved phenotypes reflect true simultaneous co-utilization or simply faster sequential consumption (diauxic or triauxic growth). Given the importance of this distinction for both mechanistic interpretation and industrial relevance, I strongly recommend including time-course measurements of glucose, xylose, and arabinose consumption for the parental strain and representative evolved strains (especially LSM isolates) under mixed-sugar conditions.

We added new experimental data on time-resolved sugar consumption profiles of the parental strain (JE3692) and the representative evolved strains (HSM1, LSM1, GXA4) under mixed-sugar conditions to Figure 3F and those of all strains to Supplementary Figure 6. These data confirmed that JE3692 exhibited co-utilization of glucose, xylose, and arabinose rather than diauxic or triauxic growth[1]. Importantly, the LSM1 isolate showed improved consumption for all three sugars while maintaining this co-utilization phenotype, indicating enhanced coordination of multiple catabolic pathways. Thus, rather than enabling co-utilization, our study demonstrates the

simultaneous optimization of multiple sugar utilization pathways. We added a paragraph interpreting the time-course data to the Results section (page 9, lines 275-282).

3) Interpretation of LSM as a general strategy for industrial applications: The carbon-limited mixed-sugar (LSM) regime appears to be the most successful at maintaining multiple pathways, yet it operates at very low total sugar concentrations (1.5 g/L). The manuscript does not sufficiently address how phenotypes evolved under such conditions would translate to industrially relevant processes, which typically involve much higher sugar loads, inhibitory compounds, and often fed-batch or continuous operation. The Discussion should more critically assess whether the generalist phenotype observed in LSM is expected to remain stable under high sugar concentrations or strong production-associated stress. Related to this, it would be useful to comment on whether high osmotic pressure in HSM (12 g/L total sugar) may itself act as a selective force. Without such discussion, the broader applicability of the findings remains uncertain.

To address this comment, we softened the language of the relevant statements in the manuscript and revised the Discussion to clarify the limitations and industrial relevance of the LSM regime (page 18, lines 548-562). Although both JE3692 and LSM1 showed reduced growth rates at elevated sugar concentrations (Supplementary Figure 10), the LSM1 strain still outperformed the parental strain in both growth rate and production (Figure 5B), supporting the view that the evolved generalist phenotype was maintained beyond the original LSM regime. Its performance under industrially relevant stresses, including pretreatment-derived inhibitors and product-associated burdens, remains to be established; nevertheless, the LSM1 strain provides a strong foundation for further development toward high-performing mixed-substrate bioprocesses. We also noted that these issues can also be addressed with additional focused ALE campaigns.

We also agree that the higher total sugar concentration in HSM (12 g/L) may have imposed an additional selective pressure through increased osmotic stress. However, our genome analysis primarily identified sugar-specific mutations rather than a clear signature of osmotic adaptation (Supplementary Information 1). We therefore consider osmotic stress a possible contributing factor, but not a primary explanation for the observed evolutionary outcome.

4) Why did GXA not yield generalists? The sequential GXA regime is conceptually the most “dynamic” condition tested, yet it did not produce generalist strains comparable to those from LSM. This is an interesting and potentially insightful negative result, but it is not sufficiently explored. One plausible explanation is that sequential exposure allows compartmentalized, sugar-specific adaptations and retention of hierarchical regulation, rather than selection for concurrent pathway optimization. The authors should expand the Discussion to explore this point. Additional evidence, such as growth or consumption profiles of GXA isolates in mixed-sugar media, would further strengthen this interpretation.

We propose that rapid substrate switching in GXA promoted compartmentalized, sugar-specific adaptation, allowing subpopulations to specialize rather than driving coordinated optimization across pathways[2]. We expanded the Discussion to address this point (page 17, lines 496-506).

Further, to address the suggestion of adding additional evidence to support this interpretation, we have added growth and sugar consumption profiles of GXA isolates in mixed-sugar conditions (Supplementary Figure 6). In addition, to more clearly represent the physiological characteristics, the volumetric sugar consumption rate (g/L/h) was replaced with the specific sugar consumption rate ($g/g_{dcw}/h$) in Figure 3E (page 9, lines 271-274).

5) Clonal isolation strategy and population-level effects: The manuscript states that clonal isolates were obtained from end-point populations, but the isolation strategy is not clearly described. It is unclear how many clones per lineage were analyzed, how they were selected, and whether they are representative of the evolved populations. This is particularly important in mixed-sugar environments, where population-level solutions or polymorphisms may arise. The authors should clarify the clonal selection procedure and discuss the possibility that some population-level benefits or trade-offs may not be captured by analyzing a single isolate per lineage.

To address this comment, we fully described the clonal isolation strategy from the endpoint populations in the Methods section (page 20, lines 610-616). Briefly, cells from the final populations were streaked onto agar plates containing the respective selection sugars, except for cells from the GXA condition, which were plated on xylose (the major improvement target). Three random fast-growing colonies were cultivated in a deep-well plate (200 µL culture volume), and the isolate demonstrating the highest overall sugar consumption was selected for downstream characterization. Therefore, the isolates analyzed in this study represent top-performing clones rather than population-average phenotypes.

We agree that this clonal selection strategy may not capture the full complexity of population-level adaptations, such as stable polymorphisms or synergistic subpopulations that can emerge in mixed-resource environments. Because our ultimate objective was to isolate a stable, high-performing generalist chassis for downstream biomanufacturing applications (e.g., indigoidine production), our screening intentionally favored individual clones with the highest independent fitness. However, we acknowledge that this approach limits our visibility into broader evolutionary dynamics or population-level trade-offs. Accordingly, we added a paragraph to the Discussion section noting this limitation and highlighting the potential for future deep-population sequencing to uncover these dynamics (page 18, lines 526-530).

6) Choice and limitations of the xylose isomerase pathway: The instability and fitness cost of the E. coli xylose isomerase pathway play a central role in the study's conclusions. While the choice of this pathway is understandable given prior work, it would strengthen the manuscript to more explicitly acknowledge alternative strategies, such as the Weimberg pathway, and discuss why the isomerase route was chosen despite its known drawbacks.

We expanded the Discussion to clearly explain the benefits and limitations of the xylose isomerase pathway, as well as the utility of alternative xylose catabolic pathways (page 18, lines 531-547). The isomerase pathway was chosen based on prior engineering strategies[1], as it enables direct entry into central metabolism via the pentose phosphate pathway (PPP). In

combination with the oxidative arabinose pathway, this design was intended to support balanced flux distribution across substrates through complementary metabolic entry points. However, since this pathway was also associated with fitness costs and instability, as reflected in our results, alternative strategies, including oxidative pathways such as the Weimberg pathway, may represent promising directions for future engineering.

7) Methodological clarity and reproducibility of ALE experiments: Key details of the ALE experiments are insufficiently described. These include culture volumes, vessel types, OD or time criteria for transfers, how sugar depletion was assessed in LSM, and how uptake rates were calculated. Given that the ALE platform is central to the study, I recommend expanding the Methods to include a dedicated subsection describing the ALE setup in a level of detail appropriate for reproducibility.

We revised the Methods section to include additional details on the ALE setup (page 19-20, lines 601-609). We also added a subsection describing how the parameters used to evaluate growth and sugar utilization were calculated (page 22, lines 701-712).

8) The manuscript raises several other interesting questions that the authors might consider addressing in the Discussion section:

8-1) Could better indigoidine production be obtained solely through the co-utilization of XYL and GLC? It appears the major contribution actually stems from ARA catabolism.

We agree that enhanced arabinose catabolism plays a key role in indigoidine production by directly supplying flux to the 2-ketoglutarate (2-KGT) node. However, our data indicate that improved glucose and xylose utilization also contributed to higher indigoidine titers, as LSM1_IND achieved higher production under mock hydrolysate conditions (24 g/L glucose, 12 g/L xylose, and 4 g/L arabinose) than under isotonic conditions (4 g/L each sugar), despite identical arabinose concentrations. This demonstrates that glucose and xylose catabolism provide additional carbon flux and metabolic energy supporting indigoidine production. Therefore, our results suggest that coordinated utilization of all three sugars, rather than reliance on arabinose alone, underpins the superior production phenotype. We have clarified this metabolic synergy in the revised manuscript (page 18, lines 531-540).

8-2) What would be the optimal industrial bioprocess scenario, considering that high sugar loads are clearly unsuitable for maintaining generalist phenotypes? Continuous systems may not be viable either, as strains tend to favor the carbon source that allows for the fastest growth and minimal metabolic burden.

Our results suggest that prolonged cultivation under high sugar loads, as well as continuous operation, is more likely to favor specialist phenotypes. In contrast, substrate-limited process configurations, such as fed-batch operation, may be more compatible with maintaining generalist phenotypes by minimizing free-sugar accumulation. These points have been incorporated into the Discussion section (page 18, lines 556-559).

8-3) Could a synthetic consortium of P. putida specialist strains outperform the current setup?

We agree that synthetic consortia represent a promising alternative strategy. However, this comparison is outside the scope of the current work and represents an important future direction for benchmarking specialist consortia against a generalist strain.

8-4) How long would the best generalist clone, LSM1, retain its beneficial properties and sugar co-utilization capacity if exposed to different selection pressures (e.g., high sugar concentrations or the stress of indigoidine production)? Would some of the top clones maintain these properties longer than others?

We agree that the long-term stability of the generalist phenotype under different selection pressures, such as high sugar concentrations or production-associated stress, is a critical consideration for industrial applications. More broadly, this is a common challenge in engineered microbial bioprocesses, as fitness trade-offs associated with burdensome heterologous pathways can drive the emergence of escape mutants during prolonged cultivation[3,4]. Although the long-term stability of the LSM1 strain under industrially relevant stresses remains to be determined and is out of the scope here, this study provides a foundation for development toward stable and high-performing industrial mixed-substrate bioprocesses.

8-5) Why was ALE focused strictly on growth rather than being linked to the production of the target metabolite(s)?

The primary objective of this study was to establish a framework for the simultaneous optimization of multiple non-native catabolic pathways, for which growth-coupled selection provides a direct and scalable proxy for metabolic performance. While biosensor-based strategies can link fitness to product formation, their implementation requires substantial additional development, including the identification of suitable sensing modules and the delicate tuning of their dynamic and operating ranges to match the production levels of the strain. Incorporating production-linked selection into ALE is a promising direction but was beyond the scope of this study.

9) Line 105-107: Please, add concentrations of sugars used individually.

We added the concentration of the individual sugars in the manuscript (page 4, lines 108-109).

10) Figure 2D: Mutations, Monsense should be nonsense.

These typos have been corrected (Figure 2D).

11) Line 108: Any explanation for the considerable reduction in growth rate?

We attribute the modestly reduced growth rate (0.46 vs. 0.60 h^{-1}) primarily to differences in cultivation conditions compared to the previous report[1]. In this study, we used an equal-mass

sugar mixture (4 g/L each) in 35 mL tubes with high agitation (1,100 rpm) using magnetic stir bars, whereas the prior study employed mock hydrolysate ratios in shake flasks with lower agitation (250 rpm). These differences in substrate composition and oxygen transfer are expected to impact *P. putida* growth kinetics. We have clarified this in the manuscript (page 4, lines 111-112).

12) Line 120-121: Please, clarify the precise criteria for OD and sugar depletion-based transfers.

Please refer to our response to Comment 7. Briefly, we revised the Methods to clarify the specific OD thresholds and the definition of complete sugar depletion in the LSM regime (page 19-20, lines 601-609).

13) Line 131-133: Clarify how these clonal isolates are selected.

Please refer to our response to Comment 5. Briefly, we clarified the clonal isolation procedure, including colony selection and screening criteria, in the Methods (page 20, lines 610-616).

14) Line 181: "motivating alternative ALE approaches" this was already part of the original hypothesis? If so, the motivation should have been the hypothesis itself.

We agree and have removed the corresponding statement from the manuscript.

15) Line 246: As mentioned previously, mixed-sugar conditions might select for a better performing population and that fact might be lost when analysing a single HSM isolate. Even if it dominates the population, the overall fitness of the whole population might be balanced by less abundant genotypes similar to real-world microbial consortia. This topic and potential limitation should be explored in the discussion.

Please refer to our response to Comment 5.

16) Line 268: I did not understand why it is "resequencing" and not just sequencing.

To avoid any ambiguity, we replaced 'resequencing' with 'whole-genome sequencing' throughout the manuscript.

17) Line 291 and 293: "HSM mixed-sugar conditions" HSM is highlighted in the caption but all conditions HSM, LSM and GXA are presented in the figure 3 D and E.

In Figures 3D and 3E, HSM, LSM, and GXA refer to ALE groups, whereas "HSM mixed-sugar conditions" in the caption was intended to describe the test condition. To avoid confusion, we have removed "HSM" from all related phrases throughout the manuscript.

18) Line 307: It would be interesting to see the effect of this mutation in Xylose and not just under Arabinose. Considering this mutation then does not appear under LSM, it seems it could be

detrimental to xylose usage. If the authors have this data perhaps would be nice to include in a supplementary figure.

While assessing the effect of the PP_3603 mutation under xylose minimal conditions may provide additional context, we note that such a single-condition growth assay would offer limited mechanistic insight into its indirect and context-dependent regulatory role. We revised the manuscript to further discuss the condition-specific effects of the PP_3603 mutation (page 11, lines 333-340). Importantly, the mutation was exclusively enriched under the ARA conditions and was not observed in the other selection regimes. This selection pattern already suggests that the mutation does not confer a fitness advantage in xylose- or glucose-utilizing conditions and may be neutral or even detrimental in those contexts. PP_3603 is a transcriptional regulator linked to glucuronate and glucarate utilization. Its putative regulon member PP_3602 shares functional annotation with *araE2*, suggesting involvement in oxidative arabinose catabolism. The phenotype, reduced growth rate but no lag phase, suggests a role in adaptation dynamics rather than direct growth enhancement.

19) Line 393-394: I appreciate the inclusion of growth profiles in C. More growth profiles should be presented earlier in the paper.

We incorporated growth and sugar consumption profiles obtained in mixed-sugar media into the revised manuscript (Figure 3F and Supplementary Figure 6).

20) Line 461-463: The first part of sentence I agree with, but then is this evolutionary set-up good for industrial and robust applications? The limitations should also be discussed.

Please refer to our response to Comment 3. We have removed this phrase from the sentence (page 17, lines 490-491) and added further discussion regarding industrial application in the Discussion section (page 18, lines 548-562).

21) Lines 541-545: Missing details on how the isolates were selected in the end of ALE.

Please refer to our response to Comment 5.

Reviewer #2 (Remarks to the Author):

I co-reviewed this manuscript with one of the reviewers who provided the listed reports. This is part of the Nature Communications initiative to facilitate training in peer review and to provide appropriate recognition for Early Career Researchers who co-review manuscripts.

We thank the reviewer for their time in evaluating our manuscript.

Reviewer #3 (Remarks to the Author):

Review of the manuscript entitled
“Simultaneous optimization of lignocellulosic sugar catabolism via systematic laboratory evolution” by Woo et al. (Control number NCOMMS-26-005036).

Adaptive laboratory evolution (ALE) has emerged as a powerful tool in metabolic engineering, both for uncovering new design principles and for improving microbial production strains. Numerous superior phenotypes have been obtained through ALE, including enhanced growth rates and product titers, expanded substrate utilization toward non-native carbon sources, and improved tolerance to environmental stresses.

Lignocellulosic biomass represents the most abundant renewable feedstock for the sustainable production of fuels and chemicals. It consists of three major polymers: cellulose (a homopolymer of glucose), hemicellulose, and lignin. Hemicellulose is structurally heterogeneous and composed of various five- and six-carbon monosaccharides, including xylose, arabinose, mannose, glucose, and galactose. Efficient and simultaneous utilization of these sugars remains a key challenge in strain engineering.

One of the central challenges in ALE is optimizing engineered microorganisms for complex feedstocks such as lignocellulosic biomass. In this context, Woo and coauthors demonstrate the application of distinct ALE regimes to evolve *Pseudomonas putida* previously engineered for xylose and arabinose catabolism. An automated ALE platform was employed to evolve the engineered strain under (i) static conditions with single selection pressures and (ii) dynamic regimes imposing selection on multiple sugars (xylose, arabinose, and glucose).

The authors show that different ALE regimes lead to distinct evolutionary outcomes, resulting in either catabolic specialists or generalists. Notably, the evolution of generalists occurred exclusively under carbon-limited mixed-sugar conditions requiring complete consumption of all three sugars. This finding provides valuable insight into how selective regimes shape metabolic breadth. Importantly, the study demonstrates that evolved generalist isolates enhanced indigoidine production relative to the parental strain, highlighting the practical relevance of the approach. The strategy also shows strong potential for extension to more heterogeneous feedstocks, such as lignocellulosic hydrolysates.

Another important outcome of the study is the observation that evolution favoured mutations that reduced metabolic burden rather than maximizing enzymatic catalytic activity. This was exemplified in strains statically evolved for improved growth on xylose as a sole carbon source. Mutations in XylB (G395R and G395W) reduced ATP-binding affinity and consequently ATPase activity. Interestingly, the wild-type ATPase activity was associated with reduced growth compared to the mutants exhibiting lower activity. These results support the authors’ conclusion that, under selective pressure, evolution prioritizes minimizing energy and resource expenditure over maintaining maximal catalytic rates.

Overall, the manuscript presents solid, carefully executed work and makes an important contribution to the development of ALE strategies aimed at improving total sugar utilization in engineered microorganisms under mixed-sugar conditions.

We thank the reviewer for the positive feedback.

1) Line 62: When strain JE3692 is first introduced, it would be helpful for a broader readership to clarify that it is a derivative of *P. putida* KT2440, a strain widely used in metabolic engineering.

We revised the text to clarify that JE3692 is a derivative of *P. putida* KT2440 and to note that KT2440 is a widely used model strain (page 2, lines 58-62).

2) Materials and Methods: The description of culture dilution during passaging is not sufficiently clear. Please specify the dilution factor used at each passage and indicate the approximate number of cells transferred (i.e., the effective bottleneck size).

We revised the Methods section to clarify the culture dilution during passaging, including the transfer volume and corresponding dilution factor. We also specified the approximate number of cells transferred by reporting the effective bottleneck sizes for each selection regime (page 19-20, lines 601-609).

3) Lines 414–426: Please refer explicitly to Supplementary Table S2 for the construction details of the plasmid expressing the indigoidine biosynthetic pathway. In line 422, please specify that plasmid pTE252_IND was transferred into JE3692 and LSM1.

We revised the text to refer to Supplementary Table 2 (Supplementary Table 3 in the revised version) for plasmid construction details (page 15, line 446) and to clarify that plasmid pTE252_IND was introduced into both JE3692 and LSM1 strains (page 15, lines 450-453).

4) Figure 4G: The legend refers to a red circle; however, only a red arrow is visible in the figure. Please clarify or correct.

The term “red circle” has been corrected to “red arrow” in the figure legend (page 15, lines 433-434).

5) Figure 5B: The concentrations of sugars used in the mock hydrolysate conditions (24 g/L glucose, 12 g/L xylose, 4 g/L arabinose) are relatively high. Could the authors clarify whether these concentrations negatively affected *P. putida* growth?

The elevated sugar concentrations used in the mock hydrolysate conditions impose a physiological burden on *P. putida*, as reflected in the reduced growth rates of both JE3692 and LSM1 at higher substrate concentrations (Supplementary Figure 10). Nonetheless, the LSM1 strain maintained superior performance relative to the parental strain under these conditions,

exhibiting both higher growth rates (Supplementary Figure 10) and enhanced production (Figure 5B). We added a discussion of this point in the revised manuscript (page 18, lines 548-555).

References

1. Elmore, J. R. *et al.* Engineered *Pseudomonas putida* simultaneously catabolizes five major components of corn stover lignocellulose: Glucose, xylose, arabinose, p-coumaric acid, and acetic acid. *Metab. Eng.* **62**, 62–71 (2020).
2. Abdul-Rahman, F., Tranchina, D. & Gresham, D. Fluctuating environments maintain genetic diversity through neutral fitness effects and balancing selection. *Mol. Biol. Evol.* **38**, 4362–4375 (2021).
3. Rugbjerg, P., Myling-Petersen, N., Porse, A., Sarup-Lytzen, K. & Sommer, M. O. A. Diverse genetic error modes constrain large-scale bio-based production. *Nat. Commun.* **9**, 787 (2018).
4. Duperray, M., Delvenne, M., François, J. M., Delvigne, F. & Capp, J.-P. Genomic and metabolic instability during long-term fermentation of an industrial *Saccharomyces cerevisiae* strain engineered for C5 sugar utilization. *Front. Bioeng. Biotechnol.* **12**, 1357671 (2024).

Responses to reviewers' comments

We sincerely thank the reviewers for their positive assessment of our revisions and for recognizing the technical strength and innovation of this work. Below, we addressed the remaining comments. Our response to each remark is presented below in blue. Changes made to the manuscript are highlighted in red.

Reviewer #1 (Remarks to the Author):

The authors have addressed most of my previous comments. The revised manuscript is substantially improved: several claims have been toned down, important missing methodological details and sugar-consumption data have been added, and the limitations of the study are now discussed more openly. Overall, I consider the work technically strong, carefully executed, and sufficiently innovative.

We thank you again for these positive evaluations.

1) That said, I still find some aspects of the manuscript somewhat overstated. The newly added data show that the improvement of the best “complex-condition” ALE isolate, LSM1, is only modest for glucose and xylose, which are the dominant sugars in most lignocellulosic hydrolysates. The strongest improvement appears to be in arabinose utilization, although arabinose usually represents a smaller fraction of such feedstocks.

We thank the reviewer for this comment, but we respectfully disagree that the improvements are modest. Specifically, as detailed in the manuscript (Page 9, Lines 273-274), the specific sugar consumption rates for xylose and arabinose in the LSM1 strain were 1.8- and 1.7-fold higher, respectively, compared to the starting strain. When integrating these increases across an entire bioprocess, a nearly two-fold increase in consumption rates for the pentose fractions (up to 40%) can have a major impact on overall process efficiency and final product titer when diverted to metabolic flux.

2) I also remain unconvinced that the clone selection strategy fully captured the potential of the evolved populations. As only three fast-growing clones per condition were screened, it is possible that better-performing or more representative generalist clones were missed.

We completely agree with the reviewer that our clone selection strategy represents a bottleneck in fully capturing the potential diversity of the evolved populations. To directly address this valid critique, we have expanded upon the limitations of our screening approach and future directions in the Discussion section.

Changes in the manuscript: We have added the following text to the Discussion (Page 18, Lines 528-534):

"Although the clone isolation strategy successfully yielded isolates with improved mixed-sugar utilization compared to the starting strain, a limitation of this study is that we did not use high-throughput screening to thoroughly explore evolved population diversity. Consequently, better-

performing or more representative generalist clones may have remained unsampled within the evolved populations. Combining ALE with high-throughput automated screening represents a promising technological advancement for future studies."

3) Some outcomes of the ALE regimes also appear relatively predictable, particularly the tendency of high-sugar mixed conditions to favor specialization toward more readily used substrates…

We appreciate the reviewer's perspective. While the emergence of specialists under high-sugar conditions (HSM) may seem predictable in hindsight or to certain experts in evolutionary dynamics, we do not believe it is universally intuitive to the broader metabolic engineering community that specialists would so consistently emerge when *all* carbon sources are abundantly present. We explicitly address the mechanisms driving the HSM condition outcomes in the Discussion section (Page 17, Lines 507-517) to ensure this dynamic—specifically how carbon abundance favors early sweeps of "sprinters"—is clearly articulated for all readers.

4) One very interesting point in the study, highlighted also by the reviewer 3#, is that evolution often selected mutations that reduced the burden of the engineered pathways, rather than mutations that made the pathways work better. In other words, the cells seem to adapt by saving energy and resources, not necessarily by becoming better at using all sugars.

This raises an important question. If ALE often works in this way, how useful are standard or here presented ALE strategies for improving complex traits such as lignocellulosic sugar co-utilization? What alternative ALE setups could help select mutations that truly improve the combined use of glucose, xylose, and arabinose (and other lignocellulosic components), instead of simply reducing cellular burden or favouring one sugar over the others? I think these questions are important and should be discussed in the Discussion/Conclusion section of the manuscript.

We thank the reviewer for raising this excellent point, which strikes at the core premise of our study. We fundamentally agree that standard, single-sugar ALE strategies are often insufficient for complex traits like co-utilization—which is exactly why we introduced the LSM regime.

In the case of our starting engineered strain, the heterologous xylose isomerase pathway imposed a massive fitness burden due to inefficient side-branch formation and background ATPase activity. The unbiased power of ALE is that it selects for the most immediate physiological relief; here, "tuning down" that severe burden was the necessary evolutionary prerequisite to achieving efficient co-utilization. Importantly, the LSM method successfully navigated this by forcing the retention and refinement of the pathways to support growth, whereas other standard regimes (like GLC or HSM) simply allowed the burdening pathway to be lost entirely via large deletions.

We agree that the broader, forward-looking question deserves explicit discussion. We have added text proposing concrete alternative or complementary strategies, including replacing cost-bearing pathway variants with lower-burden alternatives such as the Weimberg oxidative pathway, and

using burden-relieved strains such as LSM1 as starting points for subsequent ALE under more complex alternating or continuous-culture selection regimes.

We would also gently note that the premise of the study is exactly this gap: most prior ALE has used a single refined substrate, whereas real feedstocks are mixed and unrefined. Demonstrating that an appropriately designed complex-selection regime (LSM) can simultaneously optimize multiple co-utilized pathways within a single campaign — and identifying why other regimes fail to — is the central, generalizable contribution.

Changes in the manuscript: We have added the following text to the Discussion (Page 18, Lines 546-559)

"Our results highlighted the metabolic trade-offs associated with the xylose isomerase pathway, consistent with previous reports[71–73]. In this study, optimization of this pathway was constrained by intrinsic fitness costs arising from non-specific enzyme activities, which shaped evolutionary outcomes across selection regimes. More broadly, these findings indicate that when heterologous pathways impose a severe fitness burden, ALE may preferentially select for mutations that reduce this cost rather than mutations that directly improve enzyme kinetics. While the LSM strategy successfully forced the retention and coordination of these pathways rather than their complete loss, further improvements to combined sugar utilization may require complementary ALE strategies. For example, implementation of alternative oxidative routes, such as the Weimberg pathway[24,26], may enhance robustness by alleviating these trade-offs. In parallel, burden-relieved strains such as LSM1 could serve as starting points for targeted rational engineering or subsequent ALE campaigns, potentially under more complex alternating selection regimes or continuous culture setups, to shift evolutionary pressure away from basic burden reduction and toward mutations that enhance catalytic capacity and combined sugar uptake."
